# Doping Engineering of M‐N‐C Electrocatalyst Based Membrane‐Electrode Assembly for High‐Performance Aqueous Polysulfides Redox Flow Batteries

**DOI:** 10.1002/advs.202206949

**Published:** 2023-04-17

**Authors:** Bixian Chen, Huan Huang, Jiande Lin, Kailing Zhu, Le Yang, Xiang Wang, Jiajia Chen

**Affiliations:** ^1^ State Key Laboratory for Physical Chemistry of Solid Surfaces Innovation Laboratory for Sciences and Technologies of Energy Materials of Fujian Province (IKKEM) Collaborative Innovation Center of Chemistry for Energy Materials (*i*ChEM) Engineering Research Center of Electrochemical Technologies of Ministry of Education Department of Chemistry College of Chemistry and Chemical Engineering Xiamen University Xiamen Fujian 361005 China; ^2^ Beijing Synchrotron Radiation Laboratory Institute of High Energy Physics Chinese Academy of Sciences Beijing 100049 China

**Keywords:** in‐operando Raman, membrane‐electrode assembly, M‐N‐C electrocatalyst, polysulfides, redox flow batteries

## Abstract

Polysulfides aqueous redox flow batteries (PS‐ARFBs) with large theoretical capacity and low cost are one of the most promising solutions for large‐scale energy storage technology. However, sluggish electrochemical redox kinetics and nonnegligible crossover of aqueous polysulfides restrict the battery performances. Herein, it is found that the Co, Zn dual‐doped N‐C complex have enhanced electrochemical adsorption behaviors for Na_2_S_2_. It exhibits significantly electrochemical redox activity compared to the bare glassy carbon electrode. And the redox reversibility is also improved from Δ*V* = 210 mV on Zn‐doped N‐C complex to Δ*V* = 164 mV on Co, Zn‐doped N‐C complex. Furthermore, membrane‐electrode assembly (MEA) based on Co, Zn‐doped N‐C complex is firstly proposed to enhance the redox performances and relieve the crossover in PS‐ARFBs. Thus, an impressively high and reversible capacity of 157.5 Ah L^−1^ for Na_2_S_2_ with a high capacity utilization of 97.9% could be achieved. Moreover, a full cell PS‐ARFB with Na_2_S_2_ anolyte and Na_4_[Fe(CN)_6_] catholyte exhibits high energy efficiency ≈88.4% at 10 mA cm^−2^. A very low capacity decay rate of 0.0025% per cycle is also achieved at 60 mA cm^−2^ over 200 cycles.

## Graphical abstract

Due to the enhanced electrochemical adsorption and reversibility of aqueous S_2_^2−^/S^2−^, a membrane‐electrode‐assembly based on Co, Zn‐doped N‐C complex endows polysulfides aqueous redox flow battery impressive reversible capacity, high energy efficiency, and extremely low capacity decay rate in long‐term cycling. In‐operando Raman spectroscopy reveals the complicated species evolution of aqueous multi‐phase polysulfides for the first time.

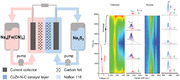

## 1 Introduction

As a large‐scale stationary energy storage technology with superior safety, redox flow batteries (RFBs) are important to improve the peak regulation capacity of renewable energy systems of photovoltaic, tidal and wind electricity grid.^[^ ^1^, ^2^ ^]^ Via the reversible electrochemical reactions between the redox couples in catholyte and anolyte, the mutual conversion of electricity, and chemical energy of redox electrolytes occurs in the rechargeable battery stack.^[^ ^3^, ^4^, ^5^, ^6^ ^]^ In this regard, redox‐active couples undoubtedly determine the performances of RFBs. Many efforts have been devoted to developing redox active electrolytes for RFBs with multiple redox electrons, structural stability and solubility, such as metallic redox ions,^[^ ^7^, ^8^ ^]^ organic molecules,^[^ ^9^, ^10^, ^11^, ^12^ ^]^ and metal‐oxo clusters.^[^ ^13^, ^14^, ^15^, ^16^, ^17^ ^]^ For example, all‐vanadium redox flow batteries (VRFBs) are one of the most mature energy storage technologies utilizing V^2+^/V^3+^ anolyte and V^4+^/V^5+^ catholyte. However, due to the limited element abundance and reversible electron number, VRFBs usually suffer from high cost (≈0.7 US dollar per Ah) and low capacity (<50 Ah L^−1^).^[^ ^18^ ^]^

Polysulfides possess low price (≈0.02 US dollar per Ah), multiple redox electrons and high solubility (8.8 M K_2_S in alkaline condition), which are potential redox active substances to construct cost‐efficient and high energy density aqueous redox flow batteries (ARFBs).^[^ ^19^ ^]^ For example, S_2_^2−^/S^2−^ is a 2‐electron reaction redox couple which will generate a high capacity of 53.6 Ah L^−1^ mol^−1^. However, due to the severe crossover of polysulfide anions, the long cycle performance of polysulfides aqueous redox flow batteries (PS‐ARFBs) is hardly comparable to those of VRFBs and Zn‐Br_2_ RFBs. For example, most of reported PS‐ARFBs delivered low Coulombic efficiencies (≈92%) and no more than dozens of cycles. Recently, a charge‐reinforced ion‐selective membrane with a polymer‐bonded chemical‐absorbing carbon layer was found to alleviate the cross contamination of polysulfides/iodide and restrain water/OH^−^ migration.^[^ ^20^ ^]^ Thereby, the as‐built polysulfide/iodide RFB can accomplish an outstanding cycle performance with an average coulombic efficiency (CE) of 99.9% at 10 mA cm^−2^.

Moreover, the inherent sluggish charge transfer kinetics of the S_2_^2−^/S^2−^ redox couples will cause severe electrochemical polarization.^[^ ^21^ ^]^ Previous cyclic voltammetry of aqueous Na_2_S_2_ solution showed poor electrochemical reversibility with a redox peak potential difference (Δ*V*) of 381 mV on the gold electrode even at a slow scan rate of 5 mV s^−1^.^[^ ^22^ ^]^ Thus, PS‐ARFBs usually suffer from very low operating current densities and energy efficiencies (EE). To facilitate redox kinetics of aqueous polysulfides, Liu designed semiconductor CoS_2_/CoS heterojunction with uneven charge distribution as robust electrocatalyst in PS‐ARFBs.^[^ ^23^ ^]^ The heterojunction‐based polysulfide/iodide ARFB delivered an EE of 71.6% at 20 mA cm^−2^ and 84.0% capacity retention for 60 cycles. On this basis, both the crossover and sluggish redox kinetics of S_2_^2−^/S^2−^ should be overcame to achieve a high‐performance PS‐ARFBs. Membrane‐electrode assembly (MEA) as a core component of fuel cell and aqueous electrolyzer systems can effectively solve multiphase material transport and electrocatalysis issues. By assembling ion exchange membrane together with catalyst layer and conductive network, MEAs exhibit enhanced high catalytic ability, large ion transfer rate and low gas permeability for high performance fuel cells and electrolysis cells. Similarly, PS‐ARFBs also need fast cation transfer and high electrocatalysis performance to insure fast electrochemical reaction kinetics, as well as low polysulfide anion permeability to guarantee long cycle performance.

In this regard, we firstly employ MOFs derived metal and nitrogen co‐doped carbon (M‐N‐C, M: Fe, Co, or Mn) based MEA to boost redox kinetics and weaken the crossover in PS‐ARFBs. MOFs derived M‐N‐C materials have drawn attention as high efficiency non‐noble metal catalyst for a series of catalytic reactions,^[^ ^24^, ^25^ ^]^ such as oxygen reduction reaction,^[^ ^26^, ^27^, ^28^ ^]^ CO_2_ reduction^[^ ^29^, ^30^ ^]^ and hydrogen evolution.^[^ ^31^ ^]^ The porous structures of M‐N‐C materials are conducive to the maximum exposure of active sites, and their regulable composition facilitates the optimization of the electronic environments. In this work, we found that the doped Co‐N_4_ in M‐N‐C complex has a higher density of the state (DOS) value than Zn‐N_4_ in the Fermi level, indicating that the addition of Co is beneficial to improve the electronic conductivity. DFT calculations also show the amount of electron transfer from Na_2_S_2_ to the Co‐N_4_ and Zn‐N_4_ are 0.56 e^−^ and 0.49 e^−^, respectively. This indicates that the appropriate doped Co‐N_4_ in M‐N‐C complex is helpful to improve its electrochemical adsorption interaction with polysulfides. In this regard, M‐N‐C electrocatalysts based MEA was firstly proposed to enhance the redox performances of PS‐ARFBs. An impressively high and reversible capacity of 157.5 Ah L^−1^ with a high capacity utilization of 97.9% could be achieved with the symmetric battery testing. Moreover, a full cell PS‐ARFB with Na_2_S_2_ anolyte and Na_4_[Fe(CN)_6_] catholyte exhibited high energy efficiency ≈88.4% at 10 mA cm^−2^. An average Coulombic efficiency of 99.7% with a considerable high capacity retention rate of 99.5% was achieved over 200 cycles. Besides, the reversibility and stability of the cathodic Na_4_[Fe(CN)_6_] and anodic polysulfides electrolytes during the charging/discharging process were further characterized systematically with in‐operando Raman spectroscopy.

## 2 Results and Discussion

### 2.1 Synthesis and Characterization of M‐N‐C Complex

**Figure 1:**
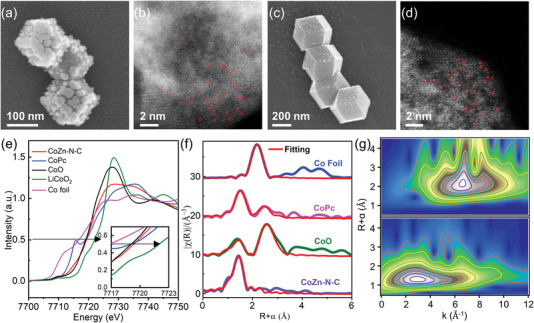
Morphological characterization and coordination structure analysis. a,c) SEM images, b,d) atomic‐resolution HAADF‐STEM images of Zn‐N‐C and CoZn‐N‐C, respectively. e) The K‐edge XANES spectra for Co foil, LiCoO_2_, CoO, CoPc, and CoZn‐N‐C. f) K‐edge FT‐EXAFS and corresponding EXAFS fitting curve in R space for Co foil, CoPc, CoO, and CoZn‐N‐C. g) WT k^2^‐weighted EXAFS contour plots of Co foil (up) and CoZn‐N‐C (down).

The ZIF‐8 was used as the precursor for the following preparation of Zn‐N‐C and CoZn‐N‐C complex via carbonizing of ZIF‐8 and Co doped ZIF‐8 nanocrystals.^[^ ^28^, ^32^, ^33^ ^]^ A SEM image of the as‐prepared ZIF‐8 material shows that it is about 350 nm in size, with a uniform rhombic dodecahedral shape (Figure S1, Supporting Information). After carbonizing at 900 °C for 1 h, the obtained nano‐sized Zn‐N‐C (**Figure** **1**a) was partially collapsed from the original rhomboid dodecahedron structure to form a porous carbon host, which was caused by evaporating of most Zn atoms from the ZIF‐8 precursor during the heating treatment. Powder X‐ray diffraction (XRD) analysis confirmed the as‐prepared Zn‐N‐C complex was amorphous (Figure S2, Supporting Information). The N_2_ adsorption/desorption and pore size distribution curves indicate that the Zn‐N‐C host has a surface area of 676 m^2^ g^−1^ and significant micro porosity (Figure S3 and Table S1, Supporting Information). Elemental mappings by energy‐dispersive X‐ray spectroscopy (EDS) (Figure S4, Supporting Information) indicate that random distributions of Zn and N elements and high‐angle annular dark‐field scanning transmission electron microscopy (HAADF‐STEM) image reveals that there is existence of single metal atoms (highlighted with red circles, Figure 1b). The final content of doped Zn amount was 14.2 wt% in Zn‐N‐C based on the results of inductively coupled plasma optical emission spectrometry (ICP‐OES, Table S2, Supporting Information).

The Co element was introduced into the ZIF‐8 host to prepare a Co, Zn co‐doped metal‐nitrogen‐carbon (M‐N‐C) complex. Via the same procedure of annealing, nano‐sized CoZn‐N‐C maintained the morphology of ZIF‐8 with 12 exposed {110} facets (Figure 1c), demonstrating an enhanced structural stability. Thus, it exhibits a specific surface area of 592 m^2^ g^−1^ and a uniform mircopore size distribution (Figure S3 and Table S1, Supporting Information). Further EDS elemental mappings (Figure S4, Supporting Information) indicate that the distribution of Co, Zn, and N elements in this complex is more evenly. As obvious illustration of HAADF‐STEM image in Figure 1d, there are also plenty of single metal atoms. Based on the X‐ray photoelectron spectroscopy (XPS) analysis (Figure S5, Supporting Information), the binding energy of metal‐nitrogrn (399.6 eV: Zn‐N for Zn‐N‐C; Co‐N or Zn‐N for CoZn‐N‐C) is closer to that of pyridinic nitrogen (400.19 eV), indicating that the main source to anchor metal atoms is more likely provided by pyridinic nitrogens. ICP‐OES results reveal that the amount of doped Co and Zn in CoZn‐N‐C was quantified to be 1.02 and 10.6 wt%, respectively. Hence, comparing with Zn‐N‐C, the doping of Co in CoZn‐N‐C will lead to the decrease of Zn content and total metal (Zn and Co) content (Table S2, Supporting Information).

To further identify the chemical state and coordination environment of Zn and Co atoms in Zn‐N‐C and CoZn‐N‐C, synchrotron X‐ray absorption spectroscopy analysis was performed. The X‐ray absorption near‐edge structure (XANES) spectra of the Zn‐N‐C and CoZn‐N‐C exhibit the Zn K‐edge position is close to the ZnPc reference (Figure S6, Supporting Information), suggesting the oxidation state of Zn in these two catalysts is Zn^2+^. The Co K‐pre‐edge of CoZn‐N‐C (Figure 1e) shows the oxidation state of Co is Co^2+^ when compared to CoO and CoPc. The pre‐edge peak of CoPc (7715 eV) is much more obvious than CoZn‐N‐C means Co^2+^ in CoZn‐N‐C has higher cation symmetry. Extended X‐ray absorption fine structure (EXAFS) spectra prove that there is no obvious metal‐metal scattering signal, comparing with Zn and Co foils (Figure S7, Supporting Information and Figure 1f). However, according to previous reports, it is intent to produce significant amount of Co nanoparticles when introducing an increasing amount of Co element.^[^ ^34^, ^35^ ^]^ Besides, the *R*‐space fittings of Zn (Figure S7 and Table S3, Supporting Information) for Zn‐N‐C and CoZn‐N‐C display dominated peaks around 1.99 and 2.02 Å. This is attributed to Zn–N coordination. While the *R*‐space fitting of Co for CoZn‐N‐C (Figure 1f and Table S4, Supporting Information) shows a dominated peak at 1.92 Å in reference to CoPc, revealing the exist of Co‐N bond. The K‐space together with wavelet transform (WT) contour plots (Figure 1g) further support the exist of Co—N bond and Co single atoms in CoZn‐N‐C. The EXAFS fittings in K space at Co K‐edge and Zn K‐edge are employed to investigate the quantitative configuration parameters in CoZn‐N‐C (Figure S8, Supporting Information). As a result, the formation of Co‐N_4_ moieties was proved in CoZn‐N‐C. Besides, each Zn atom is also coordinated with 4 N atoms in CoZn‐N‐C.

### 2.2 Fundamental Electrochemistry and DFT Calculations

**Figure 2:**
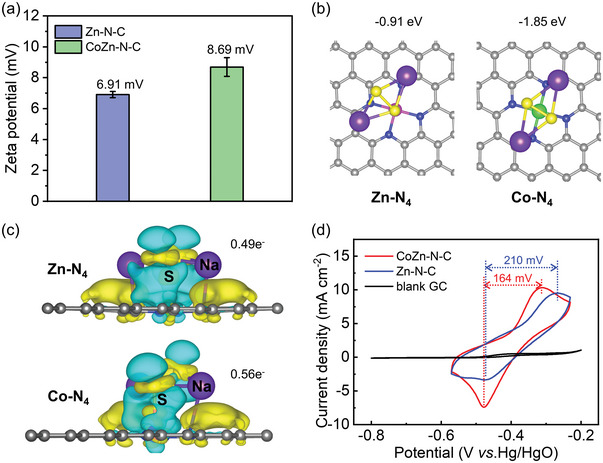
Polysulfide adsorption and cyclic voltammetry (CV) tests. a) The zeta potential of Zn‐N‐C and CoZn‐N‐C. Error bars are the standard deviation of three parallel tests. b) Calculated stable geometric configuration and c) differential charge density distribution between Na_2_S_2_ and Zn‐N_4_ (left); Na_2_S_2_ and Co‐N_4_ (right). Color codes: purple (Na), yellow (S), blue (N), gray (C), pink (Zn), and green (Co). The isosurface level is set to 0.001 e Å^−3^. The yellow and green represent gain and depletion of charges, respectively. d) CV curves of 0.1 m Na_2_S_2_ with blank glass carbon electrode (black line), glass carbon electrode coated with Zn‐N‐C (blue line) and CoZn‐N‐C on (red line).

Interestingly, zeta potential analysis reveals that the net charge of Zn‐N‐C and CoZn‐N‐C electrocatalysts are positive (**Figure** **2**a, Figure S9 and Table S5, Supporting Information). The CoZn‐N‐C shows a more positive value (+ 8.69 mV) than that of Zn‐N‐C (+ 6.91 mV), which indicates a stronger electrostatic adsorption strength toward negative polysulfides. To further probe the different electrocatalytic adsorption of Zn‐N‐C and CoZn‐N‐C, DFT simulations were conducted based on the Zn‐N_4_ and Co‐N_4_ models from the EXAFS results. The calculated results of the DOS (Figure S10, Supporting Information) show that Co‐N_4_ has a higher DOS value than Zn‐N_4_ in the Fermi level, indicating that the addition of Co is beneficial to improve the electronic conductivity of CoZn‐N‐C, thus improving the electrochemical performance. Combined with Partial DOS (PDOS) results (Figure S10, Supporting Information), it could be found that Co contributes to the DOS in the Fermi level, but Zn does not. In addition, the adsorption energy of polysulfides with stable configurations adsorbed on Co‐N_4_ and Zn‐N_4_ are simulated and illustrated in Figure 2b. The computed adsorption energies of Na_2_S_2_ on the Co‐N_4_ and Zn‐N_4_ substrates are −1.85, and −0.91 eV, respectively. Besides, the bonding characteristics between Na_2_S_2_ and Co‐N_4_ (or Zn‐N_4_) were further explored by the Bader charge analysis and differential charge density distribution (Figure 2c). The amount of electron transfer from Na_2_S_2_ to the Co‐N_4_ and Zn‐N_4_ were calculated to be 0.56 e^−^ and 0.49 e^−^, respectively. Besides, the bond lengths of S–S for Na_2_S_2_ (2.05 Å) are enlarged to 2.11 Å on being absorbed on Co‐N_4_, while it is almost remained the same on Zn‐N_4_ (2.03 Å). As a result, the stronger adsorption behaviors and larger amount of electron transfer between Na_2_S_2_ and Co‐N_4_ indicate it can weaken the S‐S bond in Na_2_S_2_ and thus reduce the decomposition barriers of Na_2_S_2_. Therefore, the appropriate addition of Co in CoZn‐N‐C will be helpful to improve its interaction with polysulfides.

Above results are further confirmed by the cyclic voltammetry (CV) test of Zn‐N‐C and CoZn‐N‐C as the electrocatalysts for aqueous polysulfides redox reactions. As shown in Figure 2d, there is no obvious redox activity of S_2_^2−^/S^2−^ on glassy carbon electrode. For Zn‐N‐C, it shows an obvious oxidation and reduction peak at −0.266 and −0.476 V versus Hg/HgO with a Δ*V* of 210 mV. As for CoZn‐N‐C, the oxidation peak is negatively shifted to −0.313 versus Hg/HgO. This indicates the redox reversibility is much improved and the Δ*V* is reduced to only 164 mV. The smaller the redox peak potential difference, the higher the electrochemical redox reversibility of the aqueous S_2_^2−^/S^2−^ will be. Meanwhile, the curves have no obvious change after 100 cycles (Figure S11, Supporting Information). Although Zn‐N‐C has a larger BET surface area (Table S1, Supporting Information), the CoZn‐N‐C electrocatalyst represents a better catalytic activity toward aqueous S_2_^2−^/S^2−^. This implies the appropriate addition of Co‐N_4_ in CoZn‐N‐C is more favorable to the redox of aqueous S_2_^2−^/S^2−^, which is consistent with the above DFT calculated results.

### 2.3 MEA‐Based Redox Flow Battery and In‐Operando Raman Characterization

**Figure 3:**
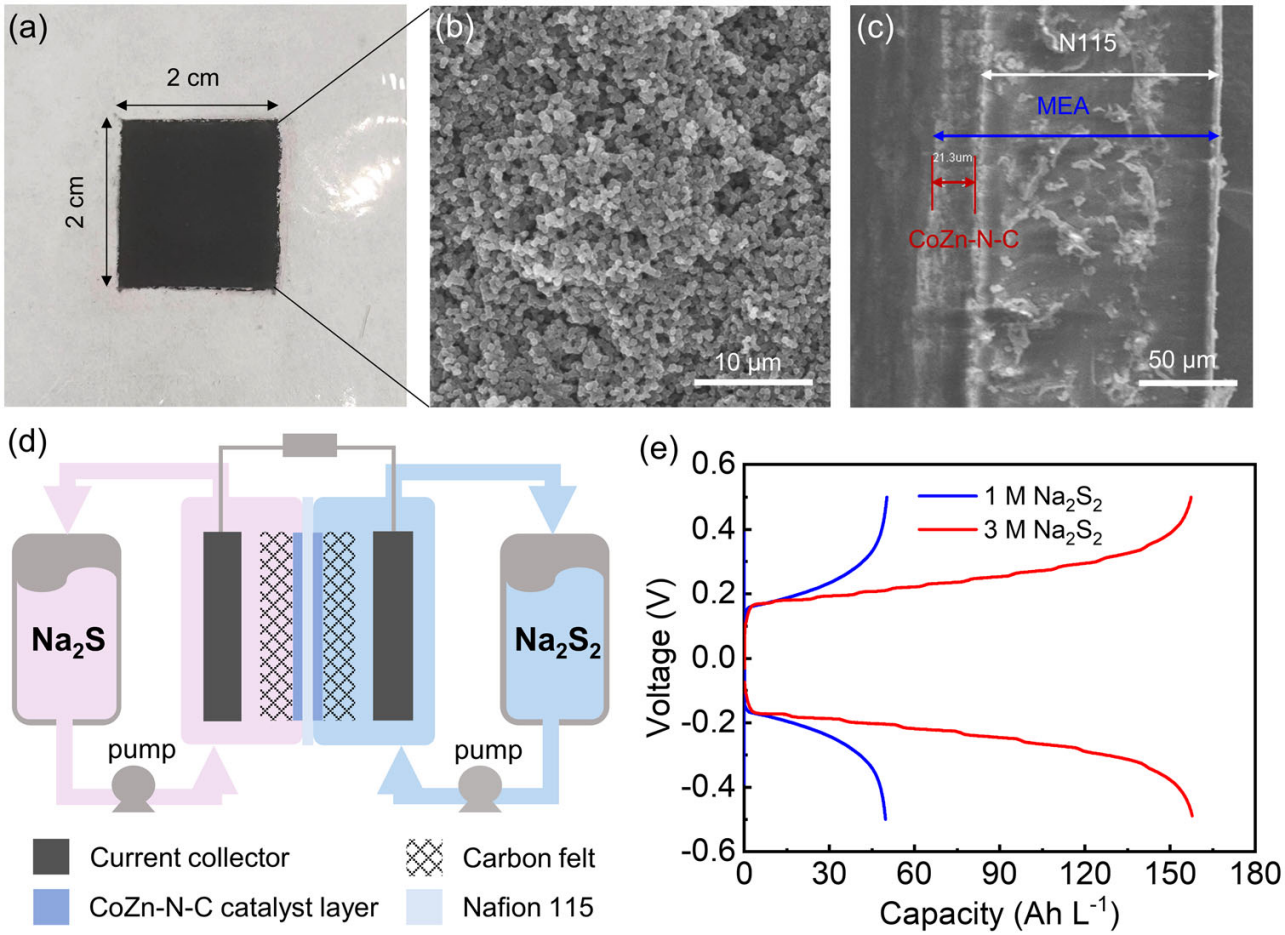
Symmetric PS‐ARFBs performances. a) Digital photo, b) front‐view SEM, and c) cross‐section SEM of CoZn‐N‐C coated Nafion membrane. d) Scheme of the symmetric PS‐ARFB. e) The charge–discharge curves of symmetric battery with 1 m Na_2_S_2_/2 m Na_2_S (in blue) and 3 m Na_2_S_2_/3 m Na_2_S (in red) at 40 mA cm^−2^.

The improved redox reversibility of aqueous S_2_^2−^/S^2−^ on the CoZn‐N‐C electrocatalyst showed a promising application in the ARFBs. CoZn‐N‐C electrocatalyst was sprayed onto the surface of Nafion 115 membrane with graphite felt to be a MEA **Figure** **3**a). The membrane has a 2*2 cm^2^ area sprayed with around 9 mg cm^−2^ CoZn‐N‐C electrocatalyst (detailed sprayed method is described in the Supporting Information). The front‐view SEM (Figure 3b) of the catalyst layer clearly shows the dodecahedral CoZn‐N‐C nanoparticles stacked compactly. In the cross‐section SEM (Figure 3c), a CoZn‐N‐C catalyst layer with a thickness of 21.3 µm was tightly attached to the Nafion 115 membrane to construct the MEA. Then, a symmetric PS‐ARFB built with the CoZn‐N‐C coated Nafion 115 membrane was used to evaluate the redox reversibility of aqueous polysulfides with M‐N‐C electrocatalyst (Figure 3d). A symmetric RFB evaluation methodology showed superiority to assess the chemical reversibility of redox couples was used in several RFB system.^[^ ^36^, ^37^ ^]^ During charging process, the cathodic Na_2_S_2_ was reduced to Na_2_S (right side), and the anodic Na_2_S was oxidized to Na_2_S_2_ (left side). Then, the generated Na_2_S in right side will be oxidized back to Na_2_S_2_ and Na_2_S_2_ (left side) will be reduced back to Na_2_S in the following discharging process. As shown in Figure 3e, there is only one charge platform which corresponds to the 2‐electron reduction process from Na_2_S_2_ to Na_2_S. Thus, 1 m Na_2_S_2_ exhibits a specific discharge capacity of 49.8 Ah L^−1^ at 40 mA cm^−2^ with the voltage cutoff at +0.5 V for charging process and −0.5 V for discharging process. This means 93.0% of the theoretical value (53.6 Ah L^−1^) can be obtained at 40 mA cm^−2^. Benefiting from the high solubility of aqueous polysulfides, the symmetric RFB can achieve an extremely high specific capacity of 157.5 Ah L^−1^ at 40 mA cm^−2^ on increasing the concentration of Na_2_S_2_ to 3 m. This indicates a high up to 97.9% capacity utilization of Na_2_S_2_ can still be retained under such high concentration. Even increasing the current density from 40 to 80 mA cm^−2^ with the same voltage cutoff at 0.5 and −0.5 V, 84.5% and 64.7% of the theoretical capacity can be still obtained, respectively (Figures S12 and S13, Supporting Information). A symmetric PS‐ARFBs without the MEA was also constructed. Due to the high electrochemical polarization on bare carbon felt, normal charging‐discharging failed at the same current density of 40 mA cm^−2^, even at a lower of 5 mA cm^−2^, within the same voltage cutoff.

**Figure 4:**
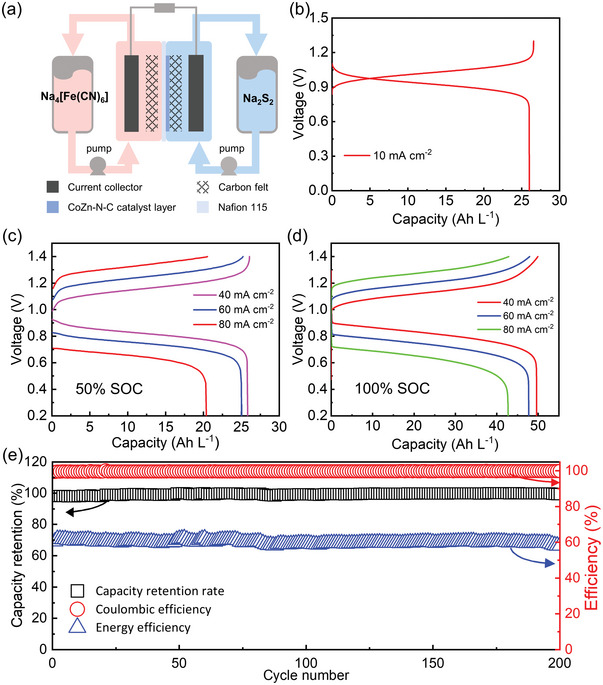
Full cell PS‐ARFBs performances. a) Scheme of full cell PS‐ARFB based on Na_2_S_2_ anolyte and Na_4_[Fe(CN)_6_] catholyte. b) The charge–discharge curve of full battery with 1 m Na_2_S_2_ and 0.5 m Na_4_[Fe(CN)_6_] at 10 mA cm^−2^. Rate performance by full charging–discharging of cell PS‐ARFB with 1 m Na_2_S_2_ and 0.5 m Na_4_[Fe(CN)_6_] at various current densities with c) 50% SOC of Na_2_S_2_ and d) 100% SOC of Na_2_S_2_. e) The cycling capacity retention, Coulombic efficiency and energy efficiency of the full cell PS‐ARFB constructed at constant current density of 60 mA cm^−2^.

The enhanced redox kinetics of polysulfides on CoZn‐N‐C also boost the electrochemical performance for the full cell PS‐ARFBs. On account of excellent stability in alkaline condition, Na_4_[Fe(CN)_6_] was chose as the cathodic active species.^[^ ^36^ ^]^ The CV curve of Na_4_[Fe(CN)_6_] catholyte exhibits a reversible redox potential at 0.365 V versus Hg/HgO (Figure S14, Supporting Information). Thus, Na_2_S_2_ and Na_4_[Fe(CN)_6_] were used as anolyte and catholyte to form a full PS‐ARFBs (**Figure** **4**a). This full redox flow battery exhibits one charging plateaus and one discharging plateaus. When constructing the RFBs with CoZn‐N‐C electrocatalyst‐based MEA at 50% state of the charge (SOC) of Na_2_S_2_, it delivers a discharging plateau at ≈0.93 V (Figure 4b). A volumetric capacity density of 26.6 Ah L^−1^ based on the anolyte can be obtained with a Coulombic efficiency (CE) of 97.9% at 10 mA cm^−2^. Taking advantage of the CoZn‐N‐C catalyst, this full cell delivered a high energy efficiency (EE) of 88.4% and voltage efficiency (VE) of 89.5%. The rate performance was also conducted at current densities ranging from 40 to 80 mA cm^−2^. As shown in Figure 4c, the full cell can deliver a discharge capacity of 25.85 Ah L^−1^ at 40 mA cm^−2^. Even increasing the current density up to 80 mA cm^−2^, more than 20 Ah L^−1^ capacity density can still be retained. Furthermore, when increasing the catholyte to make a full use of 1 m anodic Na_2_S_2_ (100% SOC, Figure 4d), a discharge capacity of 49.63 Ah L^−1^ can be achieved at 40 mA cm^−2^ which is 92.6% of the theoretical capacity of Na_2_S_2_. However, due to limited catalytic activity of Zn‐N‐C electrocatalyst, the full cell built with Zn‐N‐C electrocatalyst‐based MEA delivers poor rate performance as shown in Figures S15–S17 (Supporting Information). The galvanostatic discharge capacities of the full battery constructed with Zn‐N‐C at 60 mA cm^−2^ with 1 m Na_2_S_2_ (6 mL) and 0.5 m Na_4_[Fe(CN)_6_] (12 mL) is close to zero. Moreover, the tightly packed CoZn‐N‐C catalyst layer on Nafion membrane also exhibited apparent resist effect toward the crossover of polysulfides ions. Therefore, long‐term cycling performance of the full cell at 60 mA cm^−2^ demonstrated an excellent capacity retention ratio of 99.5% with high average CEs over 99.7% in 200 cycles. Such high capacity retention indicates a very low capacity decay rate of 0.0025% per cycle (Figure 4e). Compared to the previous reported works as summarized in Table S6 (Supporting Information), our polysulfides redox flow batteries exhibits an enhanced cycling performance and reversibility at higher current density.

**Figure 5:**
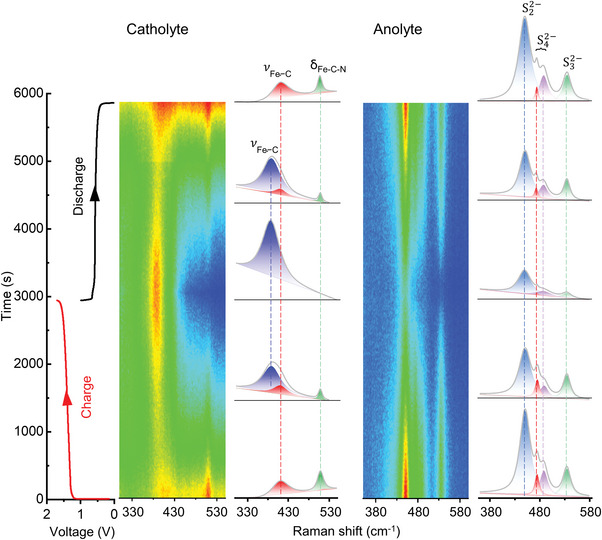
Contour map of in‐operando Raman spectroscopy of the catholyte and anolyte of the cell PS‐ARFB with full charging–discharging at a current density of 40 mA cm^−2^ and corresponding detailed Raman spectra. Catholyte: 10 mL 0.5 m Na_4_[Fe(CN)_6_]; anolyte: 11 mL 0.25 m Na_2_S_2_ in 0.25 m Na_2_SO_4_ and 0.1 m NaOH. The detail of original Raman spectra can be found in the Supporting Information.

To further prove reversibility of the catholyte and anolyte of the full cell PS‐ARFBs, in‐operando Raman spectroscopy was performed during the battery operation. 0.25 m Na_2_S_2_ in a mixed solution of 0.25 m Na_2_SO_4_ and 0.1 m NaOH was used as anolyte, and 0.5 m Na_4_[Fe(CN)_6_] was used as catholyte. During the charging process, the anodic Na_4_[Fe(CN)_6_] was gradually oxidated to Na_3_[Fe(CN)_6_]. As a result, the Fe‐C‐N bending vibration (δ_Fe‐C‐N_) at 510 cm^−1^ and stretch vibration (****ν****_Fe‐C_) of Na_4_[Fe(CN)_6_] at 410 cm^−1^ gradually weaken, as shown in the potential‐dependent Raman spectra in **Figure** **5** (left panel) and Figure S18 (Supporting Information).^[^ ^38^, ^39^ ^]^ In the meantime, there is a significant increase in the intensity of the symmetric Fe‐C stretch (****ν****_Fe‐C_) of Na_3_[Fe(CN)_6_] at 388 cm^−1^.^[^ ^40^, ^41^ ^]^ At the end of the charge process, where Na_4_[Fe(CN)_6_] is at 100% SOC, all peaks, including δ_Fe‐C‐N_ (510 cm^−1^) and ****ν****_Fe‐C_ (410 cm^−1^), assigned to Na_4_[Fe(CN)_6_] disappear and the ****ν****_Fe‐C_ (388 cm^−1^) mode belonged to Na_3_[Fe(CN)_6_] reaches a maximum. All these evolutions can go back to the original state after the discharge process, indicating the structural stability and electrochemical reversibility of the Na_4_[Fe(CN)_6_] catholyte.

The structural evolution of anodic polysulfide solution was also monitored with Raman spectroscopy in battery operation. During the charge process, the as‐prepared brown Na_2_S_2_ solution is gradually electro‐reduced and the color of the solution turned to lighter yellow. This is consistent with the in‐operando Raman characterization results in Figure 5 (right panel) and Figure S19 (Supporting Information), the peak at 451 cm^−1^ belong to *β*‐Na_2_S_2_ gradually reduced during charging process. At the same time, the characteristic peaks at 535 cm^−1^ for Na_2_S_3_ and 478 cm^−1^ for *α*‐Na_2_S_4_ was also gradually decreasing.^[^ ^42^, ^43^ ^]^ Because S^2−^ is Raman inactive, thus there is not any characteristic Raman shift of S^2−^ can be detected. The existence of a mixed solution with multiple polysulfide phases of Na_2_S_2_, Na_2_S_3_, and Na_2_S_4_ is due to the intrinsic disproportionation and hydrolysis reaction of polysulfides in aqueous solution.^[^ ^22^, ^42^, ^44^ ^]^ And because excess of polysulfide was used compared to Na_4_[Fe(CN)_6_], Na_2_S_2_, Na_2_S_3_, and Na_2_S_4_ do not disappear completely at the end of charge process. However, the following discharge process still exhibit a reversible change for these polysulfide peaks. As a result, our in‐operando Raman characterization disclose the reason of the high capacity retention rate of the full cell PS‐ARFB, which benefits from not only the high reversibility of the catholyte, but also the polysulfide anolyte.

## 3 Conclusion

In summary, atomically dispersed M‐N‐C materials were innovatively developed as electrocatalysts to enhance the redox kinetics of aqueous polysulfides. The porous 3D frame structure of M‐N‐C with large surface area, mesopores channels as well as conductive network together can promote the mass transfer and electron transfer in redox reaction of aqueous polysulfides. X‐ray absorption spectroscopy, CV measurements and DFT calculations indicated the introduced Co‐N_4_ sites can further improve the adsorb energy and boost the electrochemical redox reversibility of S_2_^2−^. Benefiting from these features, the CoZn‐N‐C electrocatalyst based symmetric PS‐ARFB delivered a very high reversible capacity of 157.5 Ah L^−1^ at 40 mA cm^−2^. A full cell PS‐ARFB achieved an impressive energy efficiency of 88.4% at 10 mA cm^−2^. Furthermore, the CoZn‐N‐C electrocatalyst based MEA drastically eliminated the crossover of polysulfides. Consequently, the CoZn‐N‐C based MEA enabled the full cell PS‐ARFB with a high average Coulombic efficiency over 99.7% and a high capacity retention rate of 99.5% over 200 cycles at 60 mA cm^−2^. Our extensive in‐operando Raman spectrum studies revealed the species evolution of aqueous multi‐phase polysulfide during charge/discharge process for the first time and verified the high reversibility of Na_4_[Fe(CN)_6_] catholyte and polysulfides anolyte in the full cell PS‐ARFB. Overall, this work innovatively employs membrane electrode assembly for high capacity and long life PS‐ARFBs and explores the in‐operando characterization techniques for advanced electrochemical energy storage systems.

## Conflict of Interest

The authors declare no conflict of interest.

## Author Contributions

J.C. proposed the project. J.C. and L.Y. wrote the manuscript. L.Y. designed the flow battery and instructed B.C. to carry out the synthesis and battery test. H.H. conducted the Synchrotron X‐ray absorption spectroscopy measurement and related data analysis. J.L. conducted the DFT calculations. L.Y conducted the in‐operando Raman characterization with the help of K.Z. and X.W. helped with the Raman analysis.

## Supporting information

Supporting Information

## Data Availability

The data that support the findings of this study are available from the corresponding author upon reasonable request.
