# Supplementary material for: Doping Engineering of M‐N‐C Electrocatalyst Based Membrane‐Electrode Assembly for High‐Performance Aqueous Polysulfides Redox Flow Batteries
Source: Adv Sci (Weinh). 2023 Apr 17;10(16):2206949. doi: 10.1002/advs.202206949 (PMC10238193; doi:10.1002/advs.202206949)
Supplement: Supplementary file 1 — Supporting Information [file ADVS-10-2206949-s001.pdf]

ADVANCED SCIENCE
Open Access

Supporting Information

for *Adv. Sci.*, DOI 10.1002/advs.202206949

Doping Engineering of M-N-C Electrocatalyst Based Membrane-Electrode Assembly for High-Performance Aqueous Polysulfides Redox Flow Batteries

Bixian Chen, Huan Huang, Jiande Lin, Kailing Zhu, Le Yang, Xiang Wang* and Jiajia Chen**

Supplementary Information

Doping Engineering of M-N-C Electrocatalyst based Membrane-Electrode Assembly for High Performance Aqueous Polysulfides Redox Flow Batteries

Bixian Chen[#], Huan Huang[#], Jiande Lin[#], Kailing Zhu, Le Yang, Xiang Wang* and Jiajia Chen**

B. Chen, J. Lin, K. Zhu, L. Yang, X. Wang, J. Chen

State Key Laboratory for Physical Chemistry of Solid Surfaces,

Innovation Laboratory for Sciences and Technologies of Energy Materials of Fujian Province (IKKEM),

Collaborative Innovation Center of Chemistry for Energy Materials (*i*ChEM),

Engineering Research Center of Electrochemical Technologies of Ministry of Education,

Department of Chemistry, College of Chemistry and Chemical Engineering,

Xiamen University, Xiamen, Fujian, 361005, China

E-mail: jiajia.chen@xmu.edu.cn; wangxiang@xmu.edu.cn; leyang0115@xmu.edu.cn.

H. Huang

Beijing Synchrotron Radiation Laboratory, Institute of High Energy Physics, Chinese Academy of Sciences, Beijing 100049, P. R. China.

Methods

Reagents and materials:

All chemical reagents including cobalt nitrate hexahydrate ($Co(NO_3)_3 \cdot 6H_2O$, ≥ 98.5%), zinc nitrate hexahydrate ($Zn(NO_3)_2 \cdot 6H_2O$, ≥ 99.0%), sulfur powder (S, ≥ 99.5%), isopropyl alcohol (C_3H_8O, ≥ 99.7%), sodium sulfate (Na_2SO_4, ≥ 99.0%), sodium hydroxide (NaOH, ≥ 96.0%), hydrogen peroxide (H_2O_2, 30%), sulfuric acid (H_2SO_4, 95.0%-98.0%), nitric acid (HNO_3, 65.0%-68.0%) and methanol (CH_3OH, ≥ 99.5%) were acquired from Sinopharm Chem. 2-methylimidazole (2-MIm, ≥ 98.0%) were from Aladdin. Sodium sulfide nonahydrate ($Na_2S \cdot 9H_2O$, ≥ 98.0%) was purchased from Acros Organics.Sodium ferricyanide ($Na_4[Fe(CN)_6 \cdot 9H_2O]$, ≥ 98%) was purchased from Sigma Aldrich. 5 wt.% Nafion solution was purchased from Du Pont Company. All chemicals were used as received without further purification.

Synthesis of Zn-N-Cand CoZn-N-C

The preparation of ZIF-8 was conducted as follow. $Zn(NO_3)_2 \cdot 6H_2O$ (2.975 g) 2-methylimidazole (3.284 g) were dissolved in 100 mL methanol with stirring, respectively. After complete dissolution, the methanol solution of dimethylimidazole was poured into the methanol solution of $Zn(NO_3)_2 \cdot 6H_2O$. Then, the mixture was stirred for 12 h at room temperature. After centrifugation, the precipitate was washed with methanol for 5 times and followed by freeze-drying overnight to obtain ZIF-8. The synthesis of Co-doped ZIF-8 was conducted as follow. $Co(NO_3)_2 \cdot 6H_2O$ (1.311 g) and $Zn(NO_3)_2 \cdot 6H_2O$ (2.975 g) were dissolved in 100 mL methanol with stirring to obtain solution A. The dimethylimidazole (3.284 g) was dissolved in 100 mL methanol with stirring to obtain solution B. The solution A and B were mixed and stirred for 12 h at room temperature. After centrifugation, the precipitate was washed with methanol for 5 times and followed by freeze-drying overnight to obtain Co-doped ZIF-8 precursors. The as-prepared ZIF-8 and Co-doped ZIF-8 precursors were respectively heated at 900 ℃ for 1 hour with a heating rate of 5 °C min^{-1} in N_2 atmosphere. After cooling, two black powders were obtained and labeled as Zn-N-C and CoZn-N-C.

Material characterizations:

The crystal phases of Zn-N-C and CoZn-N-C were identified using Powder X-ray diffraction (XRD) on a Rigaku Ultima IV diffractometer by Rigaku Japan operating at 40 kV voltage and 15 mA current with Cu Kα X-ray source. X-ray photoelectron spectroscopy (XPS) measurements were performed at Thermo Scientific ESCALAB Xi+ using Al Kα radiation as the X-ray source. Binding energies reported herein are with reference to C (1s) at 284.5 eV. The concentrations of Co element and Zn element in samples are determined by the ICP-OES (Thermofish icap 7400). The morphology of these three samples were detected with scanning electron microscopy (SEM) on a Histachi S4800 microscope at 15 kV. In addition, transmission electron microscopy TEM (HRTEM), high angle annular dark field scanning TEM (HAADF-STEM) and EDX mapping were performed on a Themis 60-300 kV transmission electron microscope (Thermofisher, America) equipped with a spherical aberration corrector of the condensor lens and Super-X detector. Co and Zn K-edge X-ray absorption near edge structure (XANES) and extended X-ray absorption fine structure (EXAFS) experiments were carried out at t 1W1B station in Beijing Synchrotron Radiation Facility (BSRF). The storage rings of BSRF were operated at 2.5 GeV with the maximum current of 450 mA. Si(111) double-crystal monochromator crystals were used to monochromatize the X-ray beam. Data reduction, data analysis were performed according to the standard procedures using the ATHENA module implemented in the IFEFFIT software packages. The quantitative curve-fittings were carried out using the module ARTEMIS of IFEFFIT. Standard procedures were used to normalize XAS data and to extract the EXAFS data from the measured absorption spectra.

Zeta potential measurement

The zeta potentials the Zn-N-C and CoZn-N-C were measured with Zetasizer Nano ZS system. 2 mg of Zn-N-C was added into 10 mL ethanol to obtain mixture A. 2 mg of CoZn-N-C was added into 10 mL ethanol to obtain mixture B. Before measurement, A

and B were ultrasonic dispersed for 1 hour, respectively. For each sample, three paralle tests were conducted.

Cyclic voltammetry test

CV curves were tested with a BioLogic VMP3 potentiostat system. A glass carbon electrode with a surface area of 0.071 cm^2 was used as working electrode, a graphite rod electrode was used as counter electrode and Hg/HgO (1 M KOH) was used as reference electrode. All the solutions were degassed with Ar for 30 min before test to remove oxygen. CV of the mixed solution (0.1 M Na_2S_2 + 0.5 M Na_2SO_4) were conducted at scan rate of 5mV s^{-1}.

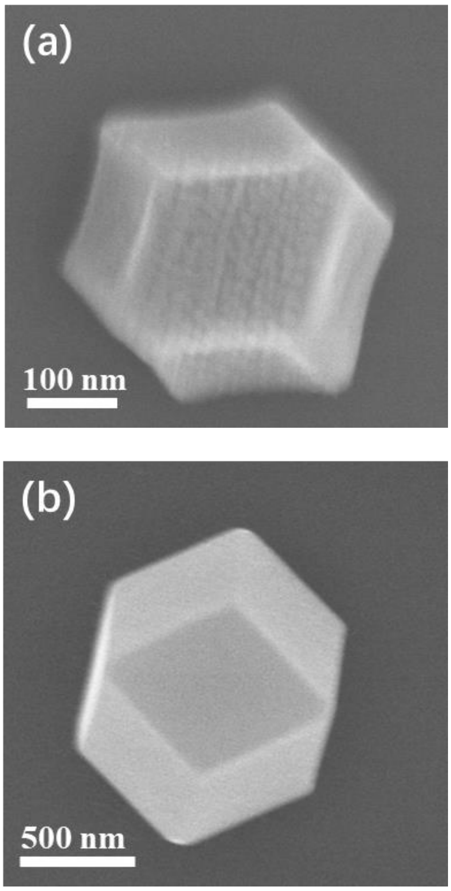

Figure S1. SEM images of (a) ZIF-8 and (b) Co-doped ZIF-8, respectively.

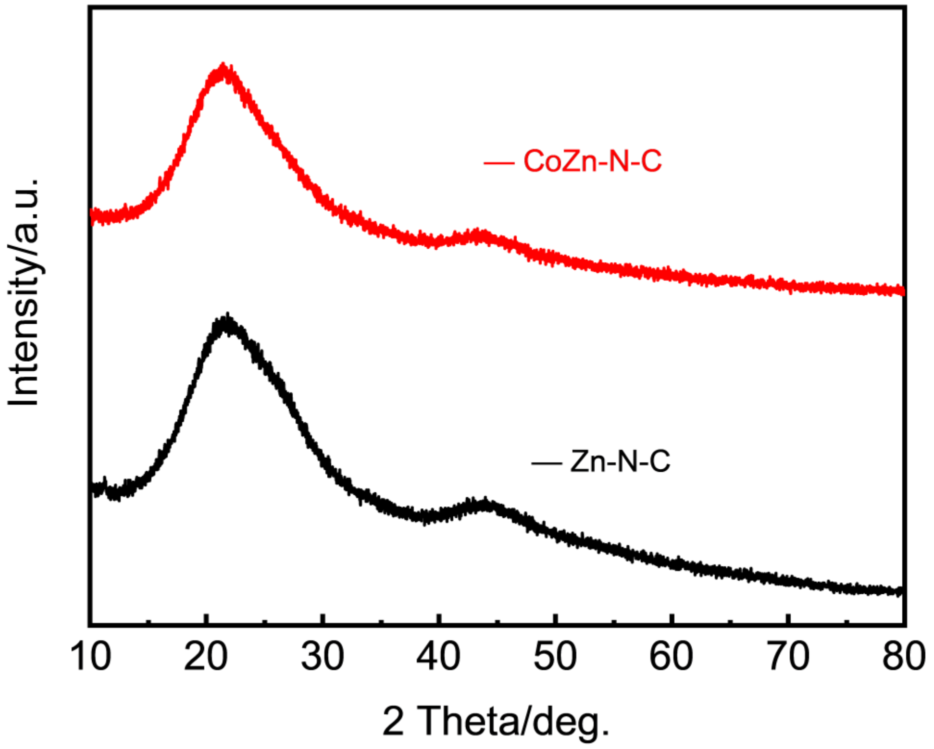

Figure S2. XRD patterns of Zn-N-C and CoZn-N-C.

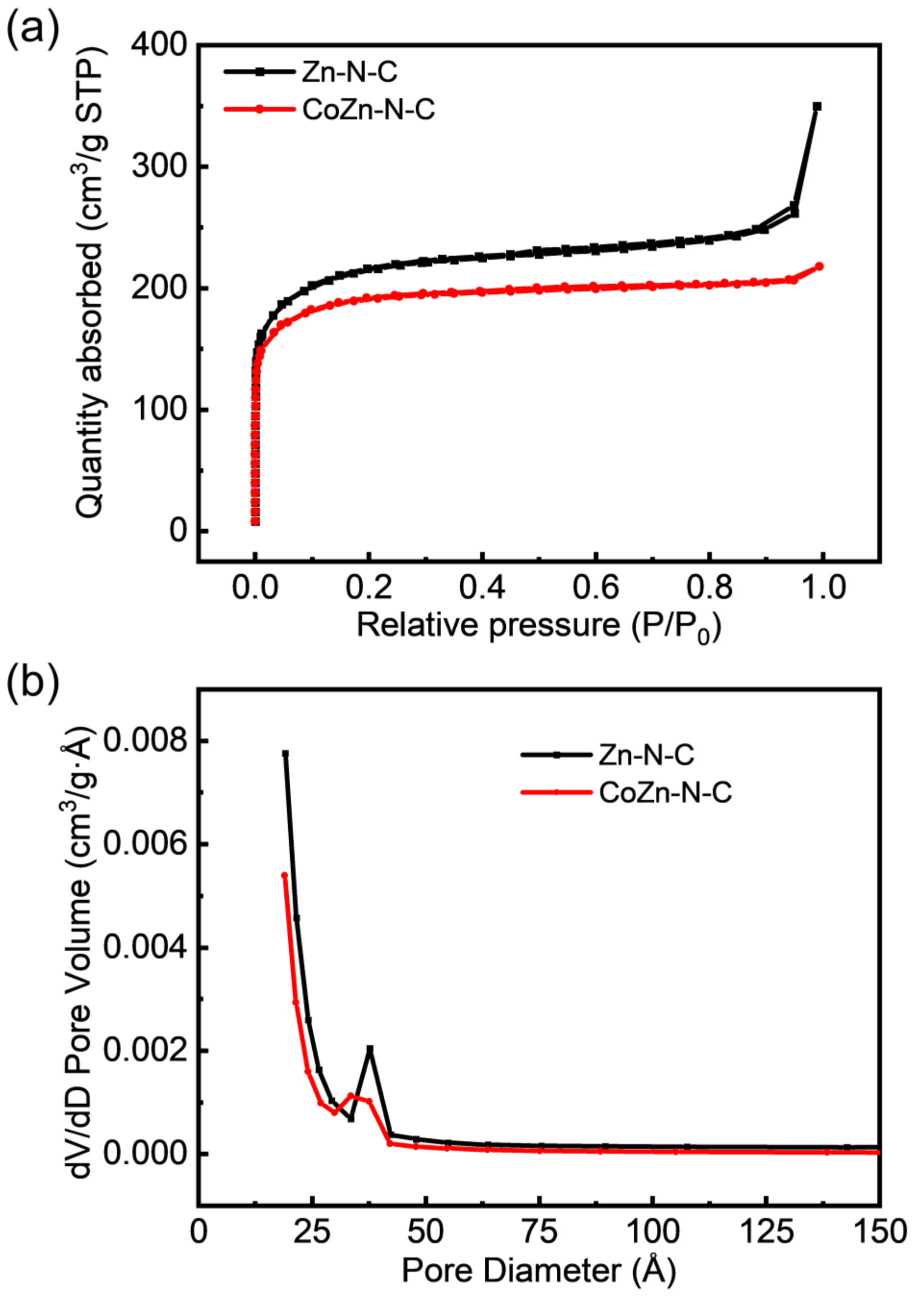

Figure S3. (a) N_2 adsorption/desorption isothermals at 77 K and (b) the pore size distribution for Zn-N-C and CoZn-N-C.

Table S1. The BET Surface Area and Pore distribution of of Zn-N-C and CoZn-N-C.

**Sample**	**BET surface area ($m^2 \cdot g^{-1}$)**	**Average pore diameter (Å)**
Zn-N-C	676.0996	32.023
CoZn-N-C	592.4450	22.749

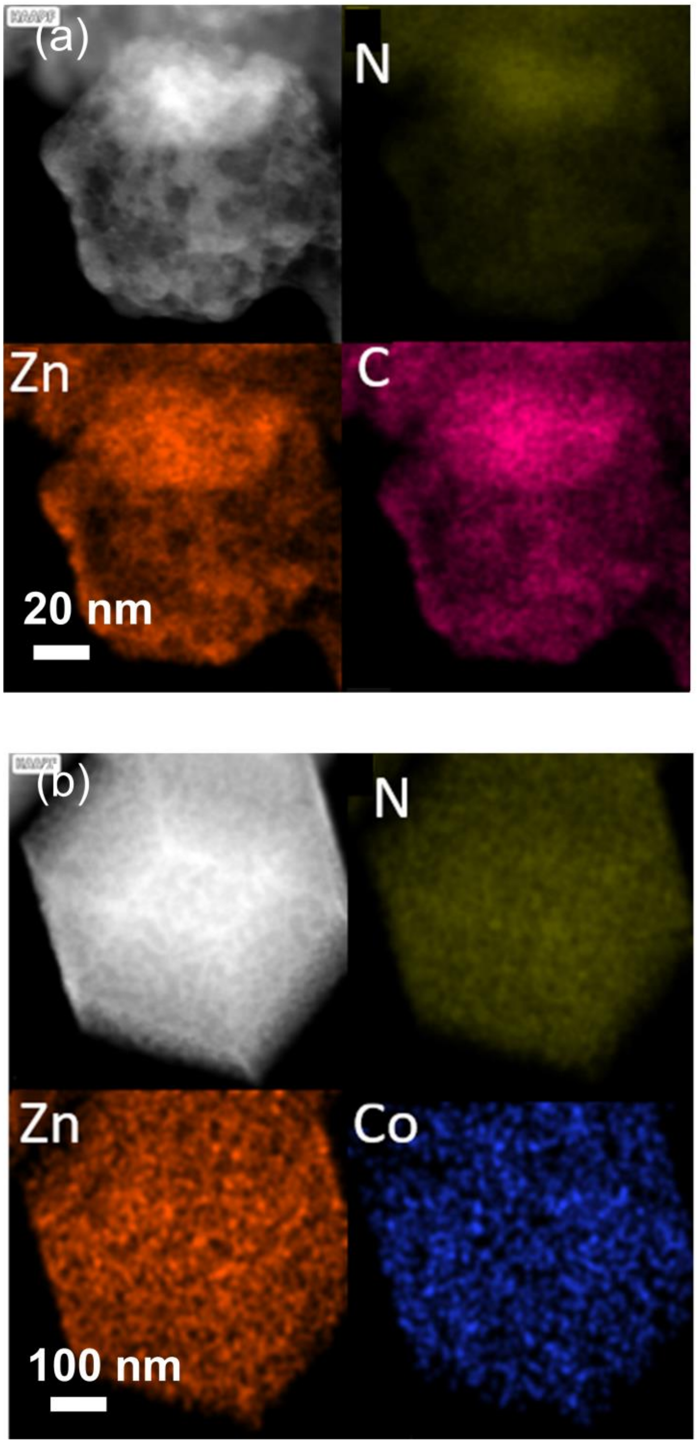

Figure S4. EDX of (a) Zn-N-C and (b) CoZn-N-C.

Table S2. ICP-OES data of Zn-N-C and CoZn-N-C.

**Sample**	**Zn（wt%）**	**Co（wt%）**
Zn-N-C	14.2	-
CoZn-N-C	10.6	1.02

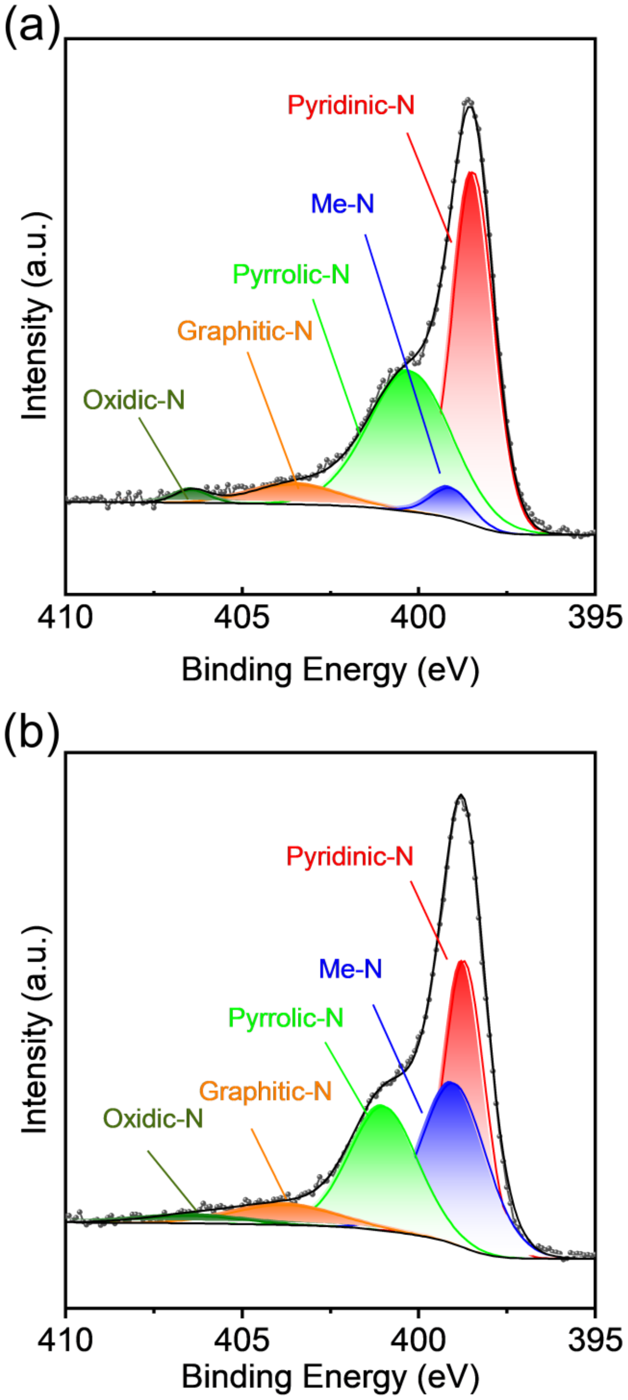

Figure S5. XPS spectra for the N 1s of (a) Zn-N-C and (b) CoZn-N-C.

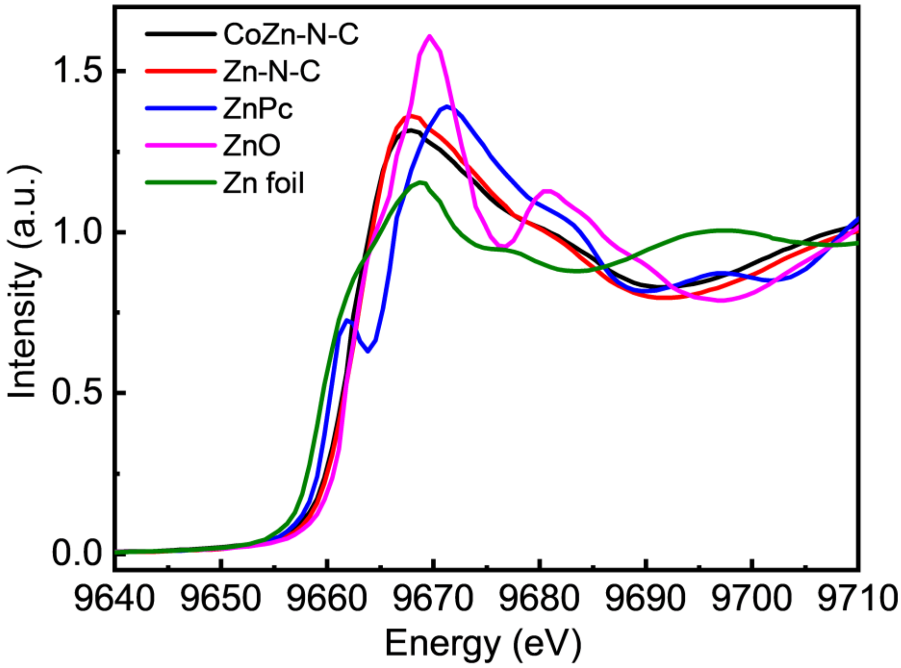

Figure S6. The Zn K-edge XANES spectra for Zn foil, ZnO, CoPc, Zn-N-C and CoZn-N-C.

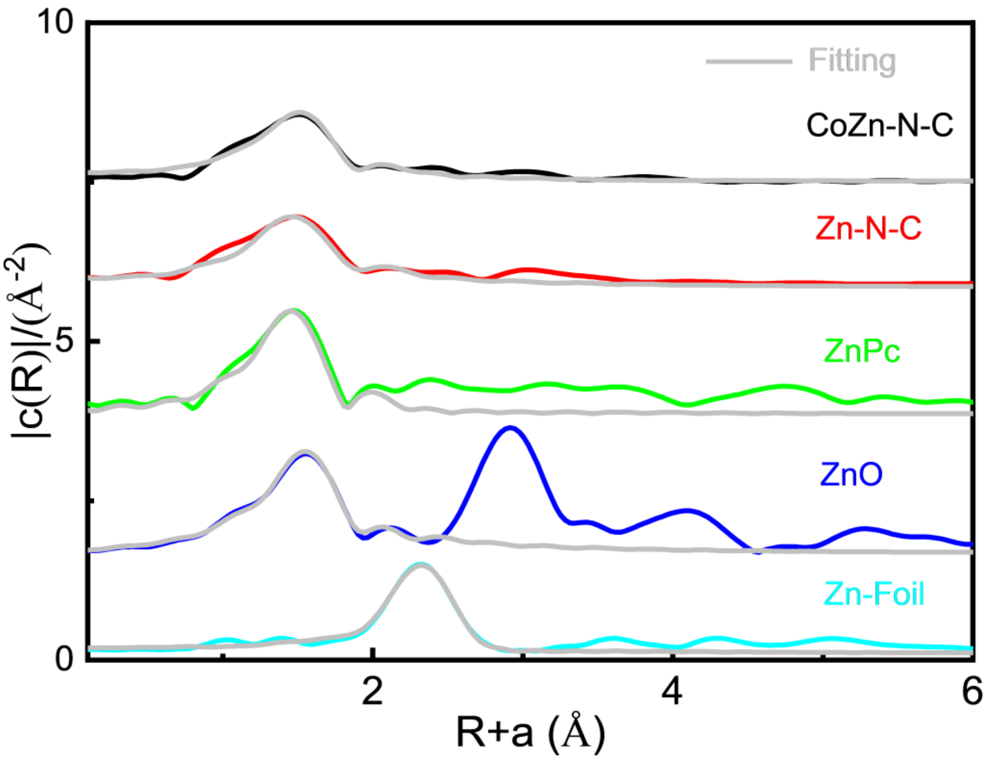

Figure S7. Zn K-edge FT-EXAFS and corresponding EXAFS fitting curve in R space for Zn foil, ZnO, CoPc, Zn-N-C and CoZn-N-C.

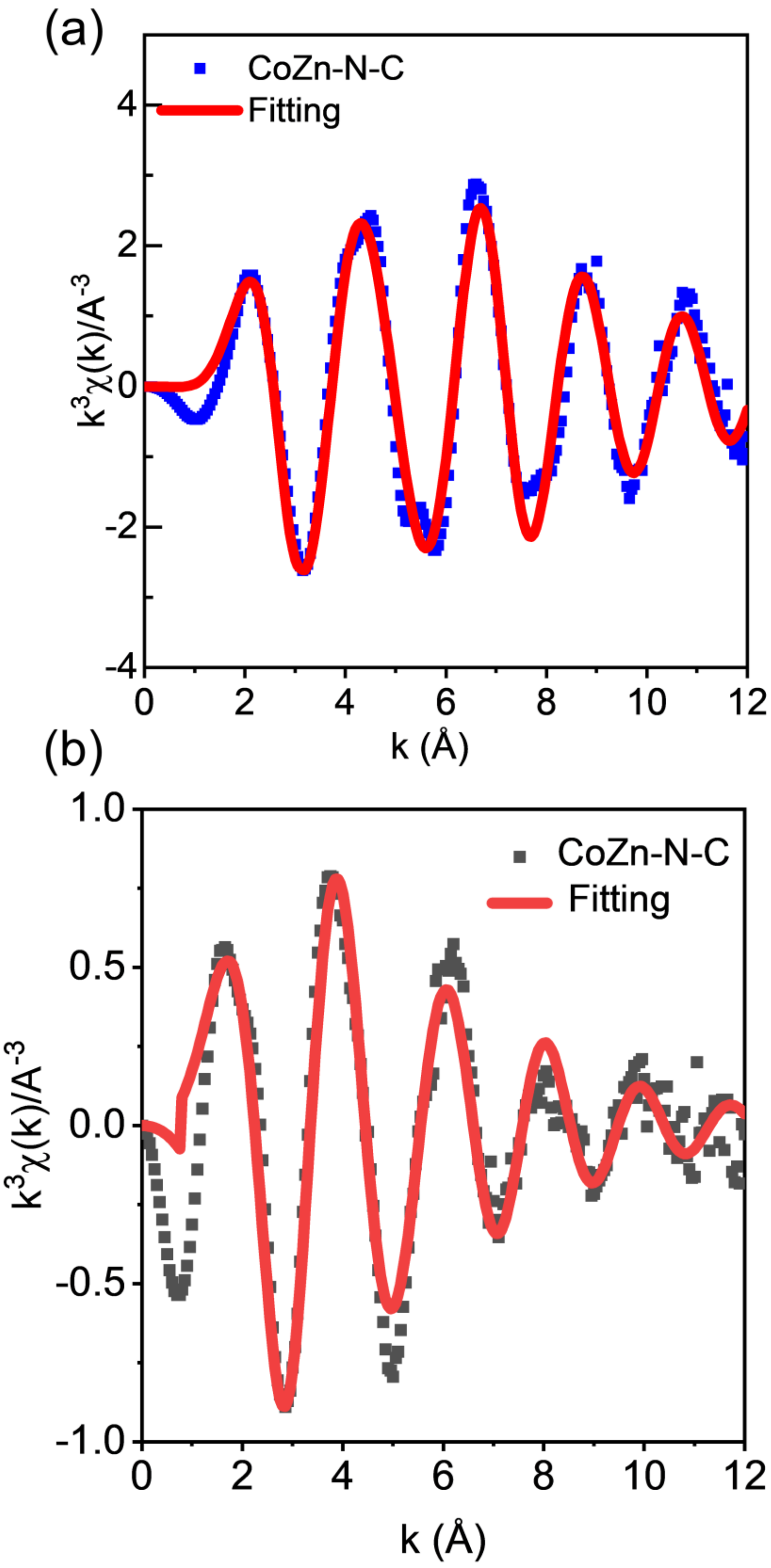

Figure S8. FT-EXAFS and corresponding EXAFS fitting curve in K space for CoZn-N-C at (a) Co K-edge and (b) Zn K-edge.

Table S3. Structural parameters of Zn-N-C and CoZn-N-C extracted from the EXAFS fitting of Zn K-edge.

**Sample**	**Path**	**N**	**R(Å)**	**σ^2 (Å^2)**	**ΔE_0 (eV)**	**R factor**
**Zn-Foil**	Zn-Zn	6[a]	2.64(2)	0.011(3)	0.1	0.007
**ZnO**	Zn-O	4[a]	1.96(0)	0.004(3)	4.8	0.003
**ZnPc**	Zn-N	4[a]	1.97(0)	0.003(3)	2.8	0.013
**Zn-N-C**[b]	Zn-N	4.2	1.99(3)	0.006(3)	1.6	0.006
	Zn-O	0.8	2.13(1)	0.005(3)		
**CoZn-N-C**[b]	Zn-N	**4.1**	2.02(1)	0.007(1)	2.4	0.020

N:coordination number;

R: distance between absorber and backscatter astoms;

σ^2: the Debye-Waller factor value;

ΔE_0 (eV): inner potential correction to account for the difference in the inner potential between the sample and the reference compound.

R factor indicates the goodness of the fit.

Data ranges of ZnO, ZnPc, CoZn-N-C and Zn-N-C: $2.5 \leqslant k \leqslant 12.5$ Å^{-1}, $0.75 \leqslant R \leqslant 2.0$ Å.

Data ranges of Zn-Foil: $2.9 \leqslant k \leqslant 12.8$ Å^{-1}, $1.6 \leqslant R \leqslant 2.9$ Å.

[a]Ns for Zn-Foil, ZnO, ZnPc were set to fixed values according to their crystal structures.

[b]S_0^2 for CoZn-N-C and Zn-N-C were fixed as 0.997 coming from the fitting result of ZnPc.

Table S4. Structural parameters of CoZn-N-C extracted from the EXAFS fitting.

Sample	Path	N	R(Å)	σ^2 (Å^2)	ΔE_0 (eV)	R factor
**Co-Foil**	Co-Co	12[a]	2.49(1)	0.006(1)	-4.3	0.004
**CoO**[a]	Co-O	6 [a]	1.97(1)	0.017(3)	-1.0	0.017
	Co-Co	6 [a]	2.97(1)	0.007(1)		
**CoPc**[b]	Co-N	4 [a]	1.91(2)	0.003(1)	7.9	0.011
	Co-C	8 [a]	2.94(1)	0.003(1)		
	Co-N	16 [a]	3.12(1)	0.014(1)		
**CoZn-N-C**[b]	Co-N	**4.2**	1.92(1)	0.007(1)	-1.8	0.014
	Co-C	3.8	2.82(4)	0.017 (2)		

N:coordination number;

R: distance between absorber and backscatter astoms;

σ^2: the Debye-Waller factor value;

ΔE_0 (eV): inner potential correction to account for the difference in the inner potential between the sample and the reference compound.

R factor indicates the goodness of the fit.

Data ranges of Co-foil, CoO and CoZn-N-C : 2.5≤k≤12.5 Å^{-1}, 1.2≤R≤3.0 Å.

Data ranges of CoPc: 3.0≤k≤11.0 Å^{-1}, 1.0≤R≤2.9 Å.

[a]Ns for Co-foil, CoO, CoPc were set to fixed values according to their crystal structures.

[b]S_0^2 for CoZn-N-C was fixed as 0.798 coming from the fitting result of CoPc.

(a)

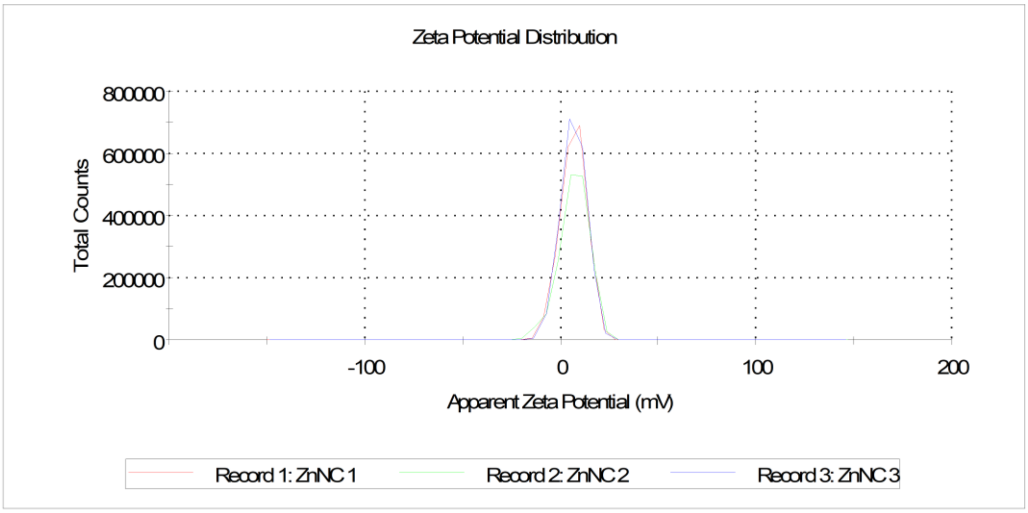

(b)

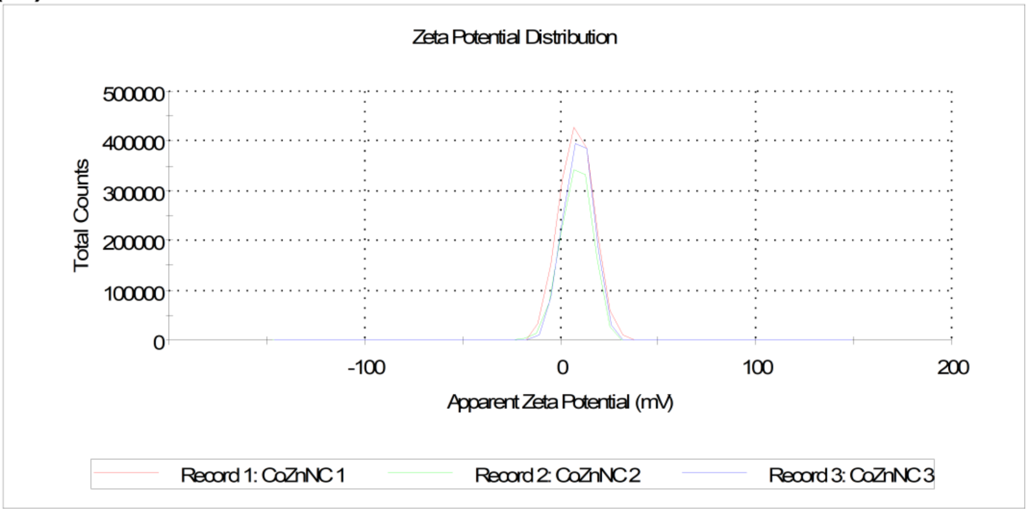

Figure S9. Zeta potential of (a) Zn-N-C and (b) CoZn-N-C.

Table S5. Zeta potentials data of Zn-N-C and CoZn-N-C for three paralle tests.

Sample	Zeta potential (mV)	Zeta potential (mV)	Zeta potential (mV)	Mean of zeta potential (mV)	Standard deviation
Zn-N-C	6.80	7.14	6.79	6.91	0.199
CoZn-N-C	8.32	8.36	9.39	8.69	0.607

Theoretical Calculation

All of the spin-polarized first-principle calculations were carried out using the Vienna *ab* initio Simulation Package (VASP, version 5.4.4) code [1-2] within the framework of density functional theory (DFT). The projector augmented wave (PAW) [3] pseudopotential was applied to describe electron core interactions, and the Perdew-Burke-Ernzerhof (PBE) [4] function of the generalized gradient approximation (GGA) [5] was used to describe exchange-correlation potential. The models of CoN_4 and ZnN_4 (71 atoms) were built by depositing CoN_4 or ZnN_4 moiety into 6×6 supercells of graphene with vacuum layer of 20 Å, respectively. In all calculations, a cutoff energy of 500 eV was used, and the convergence criteria were 10^{-6} eV and 0.01 eV/Å for electron energy and ions force, respectively. Additionally, Grimme's DFT-D3 method [6] was used to include the physical van der Waals interactions. A *k*-point mesh of 3×3×1 Monkhorst Pack for geometric optimization and 9×9×1 Monkhorst Pack for the density of states (DOS) calculation were in the Brillouin Zone were sampled.[7] The adsorption energies (E_{ads}) of polysulfides adsorption on substrates (CoN_4 or ZnN_4) were calculated by

$$E_{\text{ads}} = E_{\text{polysulfides@substrates}} - \left(E_{\text{substrates}} + E_{\text{polysulfides}}\right)$$

where $E_{\text{polysulfides@substrates}}$, $E_{\text{substrates}}$ and $E_{\text{polysulfides}}$ are the total energies of the substrates adsorbed with the molecules, pristine substrate slabs, and the isolated molecules, respectively. Bader charge analysis[8] was employed to study the charge transfer between the polysulfides and the substrates. We perform the differential charge density analysis by the following equation

$$\Delta\rho = \rho_{\text{polysulfides@substrates}} - \rho_{\text{substrates}} - \rho_{\text{polysulfides}}$$

where $\rho_{\text{polysulfides@substrates}}$, $\rho_{\text{substrates}}$, and $\rho_{\text{polysulfides}}$ represent the charge densities of the CoN_4 or ZnN_4 with polysulfides, pristine CoN_4 or ZnN_4, and the polysulfides, respectively. The VESTA software[9] was used to depict all the atomic models and differential charge density distribution.

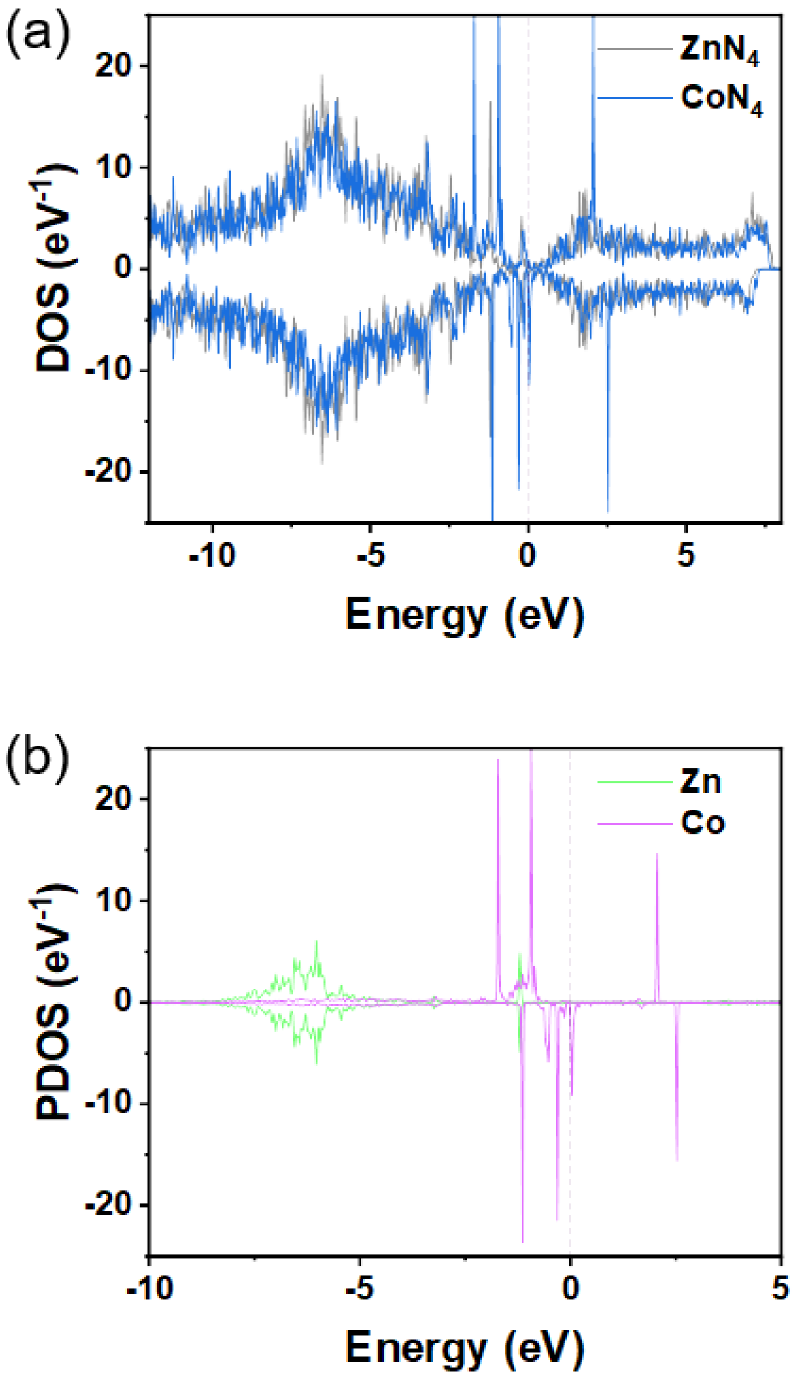

Figure. S10. (a) Density of state curves of Zn-N_4 and Co-N_4. (b) Partial density of state curves of Zn in Zn-N_4 and Co in Co-N_4.

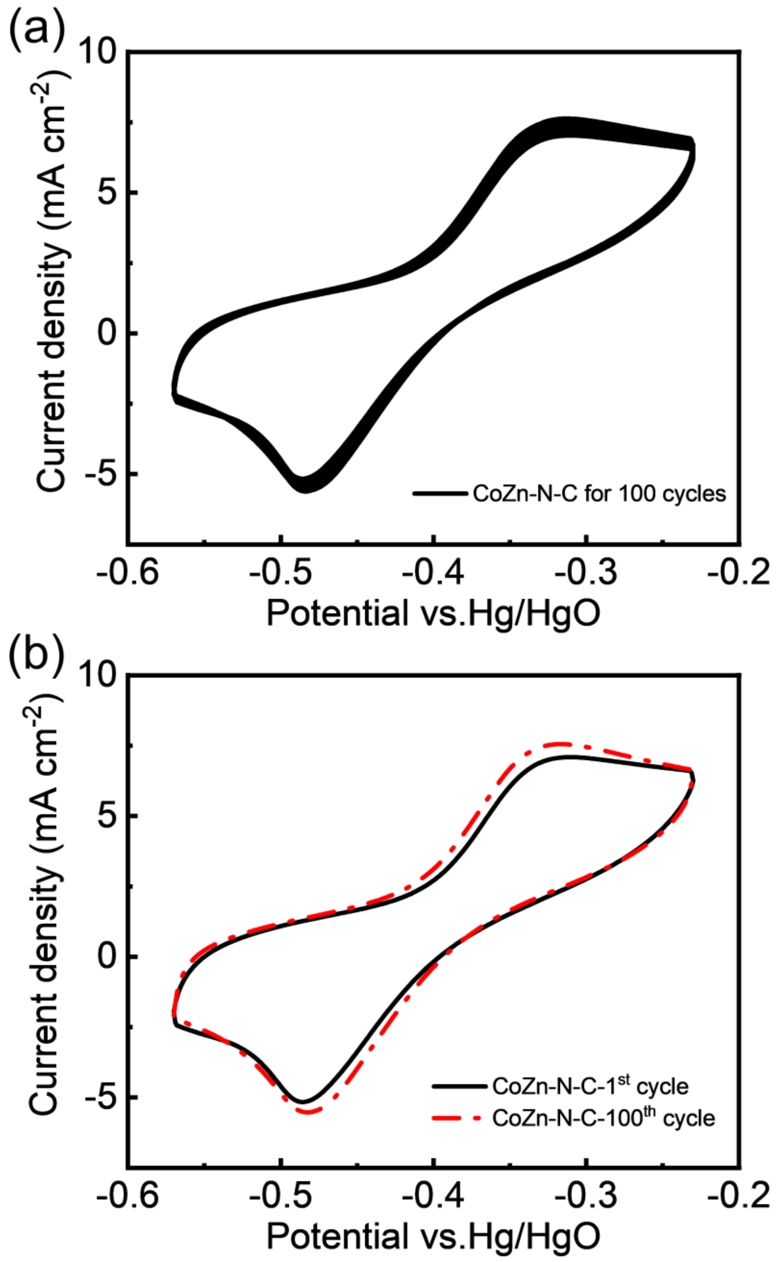

Figure S11. CV curves of 0.1 M Na_2S_2 with CoZn-N-C at 5 mV s^{-1} for (a) 100 cycles, (b) the first and the 100th cycles.

Assembly of Redox Flow Battery

Two endplates were made stainless steel. Two titanium plates with a 2 * 2 cm^2 sinking plane were used as bipolar plates. The Nafion 115 membrane was pretreated with 5 wt% H_2O_2 under 80℃ for 30 min and followed by boiling in 1M H_2SO_4 at 80℃ for 30 min. The carbon felt was boiled in mixed solution (H_2SO_4:HNO_3=3:1 v/v) for 3 hours. Then, the Nafion membrane and carbon felt were soaked in 1 M NaOH overnight and washed with DI water. For symmetric PS-ARFB, both sides of the Nafion membrane were sparied with ink 1. The ink 1 is a mixture of 37.5 mg CoZn-N-C, 7 mg PVDF, 0.5 mL DMF and 15 mL IPA. Two pieces of carbon felt were loaded with ink 2. The ink 2 is a mixture of 100 mg CoZn-N-C, 8 mg PVDF, 1.5 mL DMF and 40 mL IPA. For full cell PS-ARFB, one side of the Nafion membrane were sparied with ink 1. The ink 1 is a mixture of 37.5 mg CoZn-N-C, 7 mg PVDF, 0.5 mL DMF and 15 mL IPA. A piece of carbon felt was loaded with ink 2. The ink 2 is a mixture of C 100 mg CoZn-N-C, 8 mg PVDF, 1.5 mL DMF and 40 mL IPA. Another carbon felt do not support any catalysts and used directly after alkaline treatment. The symmetric and full cell PS-ARFBs were assemblied with above accessories.

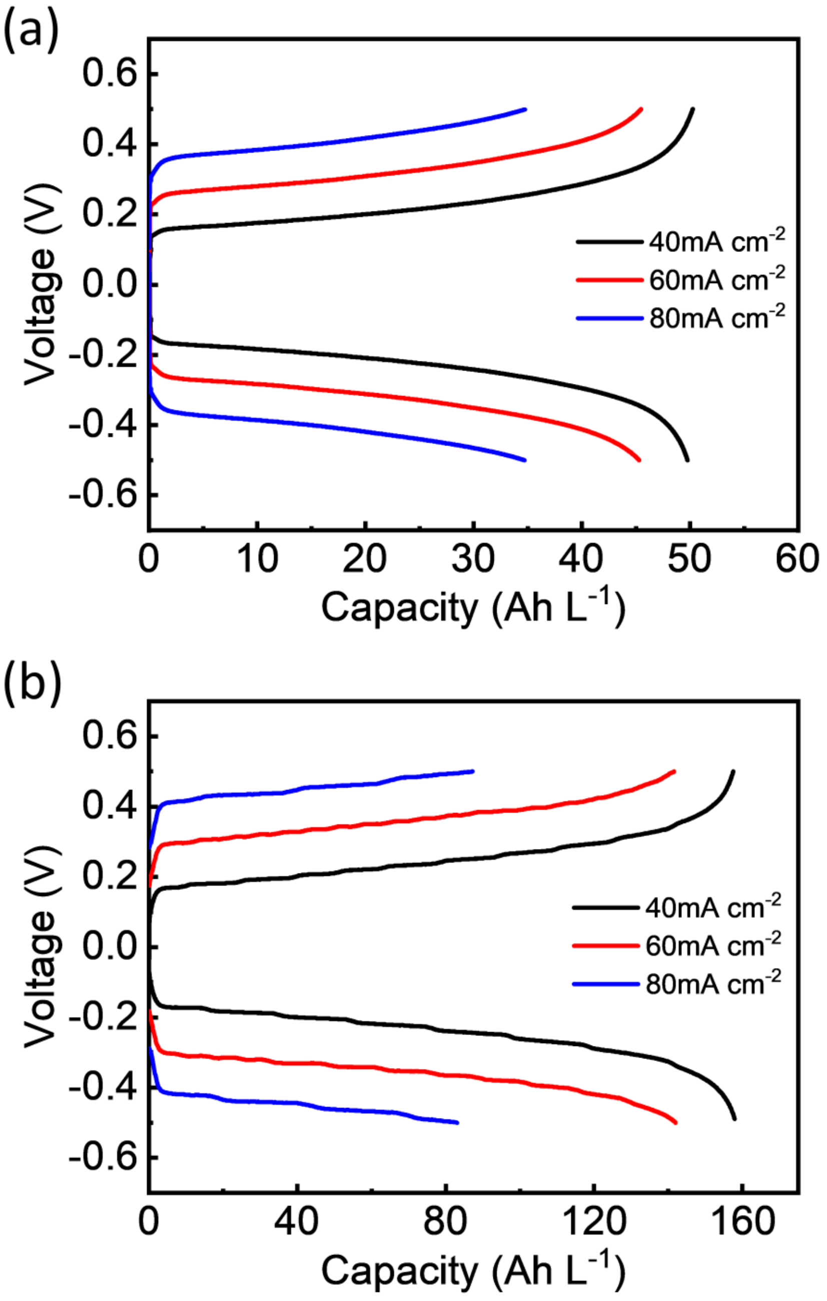

Figure S12. The charge-discharge curves of symmetric battery constructed with CoZn-N-C with (a) 1M Na_2S_2/2M Na_2S and (b) 3M Na_2S_2/3M Na_2S at 60 and 80 mA cm^{-2}.

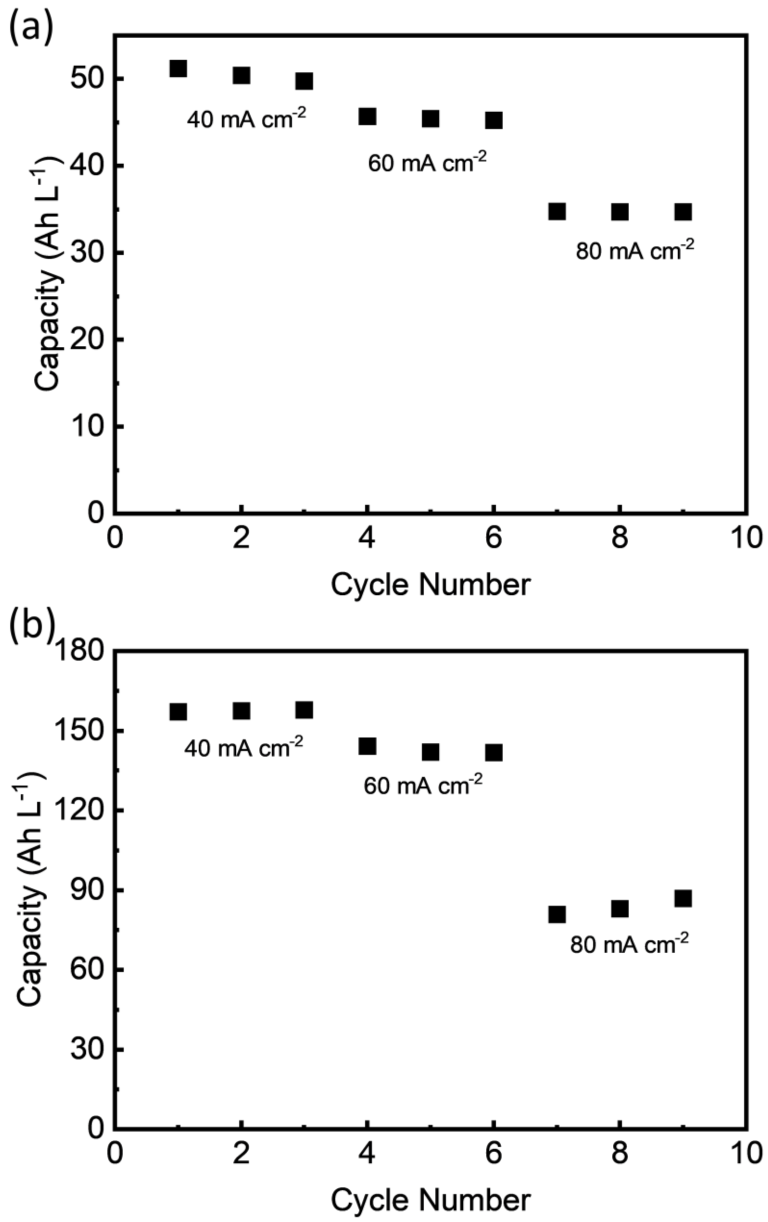

Figure S13. The galvanostatic discharge capacity of the symmetric battery constructed with CoZn-N-C at 40 mA cm^{-2}, 60 mA cm^{-2} and 80 mA cm^{-2} with (a) 1M Na_2S_2 (6 ml) and 2 M Na_2S (6 ml); (b) 3 M Na_2S_2 (6 ml) and 3 M Na_2S (12 ml).

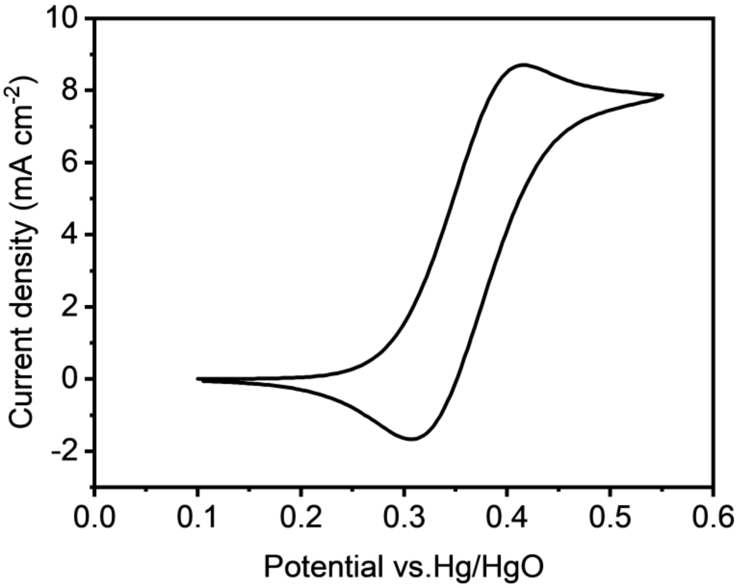

Figure S14. CV curves of 0.1 M $Na_4[Fe(CN)_6]$ in 0.5 M Na_2SO_4 with pure glass carbon electrode (scan rate: 5mV s^{-1}).

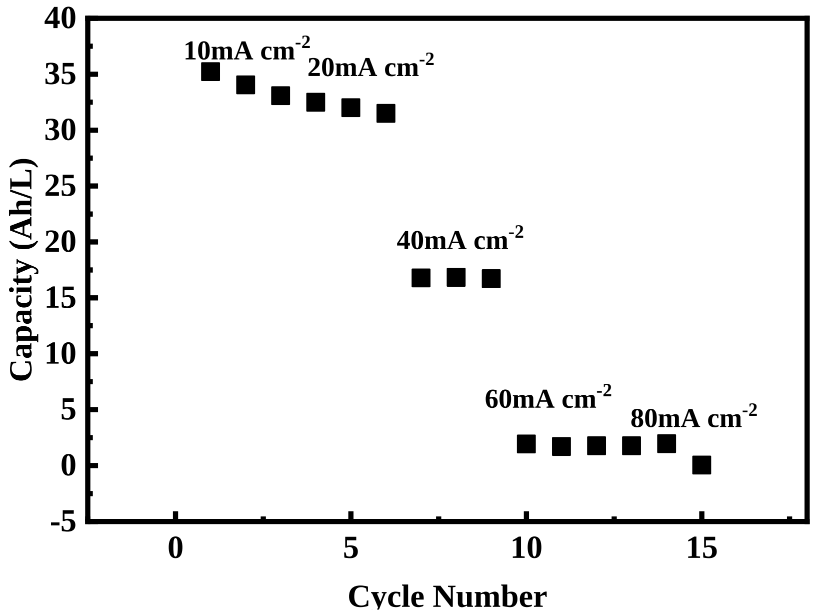

Figure S15. The galvanostatic discharge capacity of the full battery constructed with Zn-N-C at 10 mA cm^{-2}, 20 mA cm^{-2}, 40 mA cm^{-2}, 60 mA cm^{-2}and 80 mA cm^{-2} with 1M Na_2S_2 (6 ml) and 0.5 M $Na_4[Fe(CN)_6]$ (12 ml).

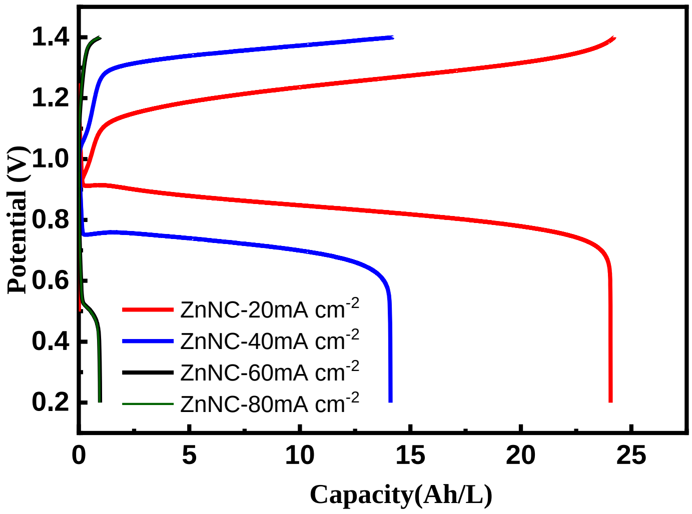

Figure S16. Rate performance by full charging-discharging of cell PS-ARFB constructed with Zn-N-C at 20 mA cm^{-2}, 40 mA cm^{-2}, 60 mA cm^{-2} and 80 mA cm^{-2} with 1M Na_2S_2 (6 ml) and 0.5 M $Na_4[Fe(CN)_6]$ (12 ml).

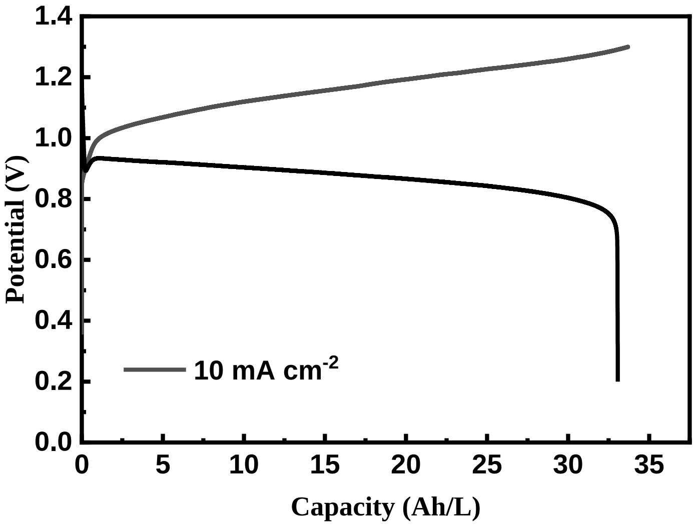

Figure S17. The charge-discharge curves of symmetric battery constructed with Zn-N-C with 1M Na_2S_2 (6 ml) and 0.5 M $Na_4[Fe(CN)_6]$ (12 ml) at10 mA cm^{-2}.

Table S6. Battery performances of reported aqueous polysulfide flow batteries.

Catholyte	Anolyte	Number of cycles: capacity retention rate	Average CE	Reference
0.5 M $Na_4[Fe(CN)_6]$	1 M Na_2S_2	200 cycles: 99.5%<br>60 mA cm^{-2}	99.7%	This work
4 M KI	3 M K_2S_2	50 cycles: > 98%	93%	Ref [10]
2 M NaI	2 M Na_2S_2	60 cycles (20 mA cm^{-2}): 84%	> 91%	Ref [11]
4 M KI	2 M K_2S_2	10 mA cm^{-2}<br>500 cycles: no detectable capacity decay	99.9%	Ref [12]
2 M NaI	2 M Na_2S_2	20 mA cm^{-2}<br>50 cycles: 87%<br>20 cycles: 92%	92%	Ref [13]
2 M NaI	2 M Na_2S_2	60 cycles (20 mA cm^{-2}): 87%<br>80 cycles (40 mA cm^{-2}): 87%	95%	Ref [14]
1 M $NaClO_4$+EC: PC+5% FEC	0.25 M Na_2S_4+0.1 M NaOH	100 cycles (0.5 mA cm^{-2})<br>200 cycles (2 mA cm^{-2})	N/A	Ref [15]
0.75 M NaI+0.25 M NaI_3+0.5 M Na_2SO_4	0.5 M Na_2S_2+0.25 M Na_2S_4+1 M Na_2SO_4	50 cycles (20 mA cm^{-2}):<br>VE retention rate =90.91%	N/A	Ref [16]
1 M Na_2S_2+0.1 M NaOH	PSB-1 M Br_2+2 M NaBr;<br>PSI-1.5 M NaI+0.5 M Na_2SO_4	1.5 mA cm^{-2}<br>PSB: 50 cycles<br>PSI: 100 cycles	PSB: 98.6%<br>PSI: 98.7%	Ref [17]

In-operando Raman measurement for the full cell PS-ARFB.

The Raman spectum was collected with a confocal Raman microscope (Alpha-300, WITec) with an excitation wavelength of 532 nm every 10 s during the discharging-charging process of the RFB. A 50 μm multimode fiber was used for Raman signal collection and also as a confocal hole. The signal was then sent via an optical fiber port to a UHTS-300 spectrometers (Witec) equipped with an EMCCD detector (1600 × 200 pixels, Newton, Andor) to achieve the highest sensitivity. During the charging/discharging of the full cell PS-ARFB, the anolyte and catholyte were flowing through a quartz flow cell with an optical path of 1 mm under a microscope objective. The microscope objective was of 20× magnification with a numerical aperture of 0.45. An edge filter was equipped to filter the exciting line, and an 1800 g/mm grating was employed for the experiments. The background of denoised spectra is removed by the auto-adaptive background subtraction of the used quartz flow cell.

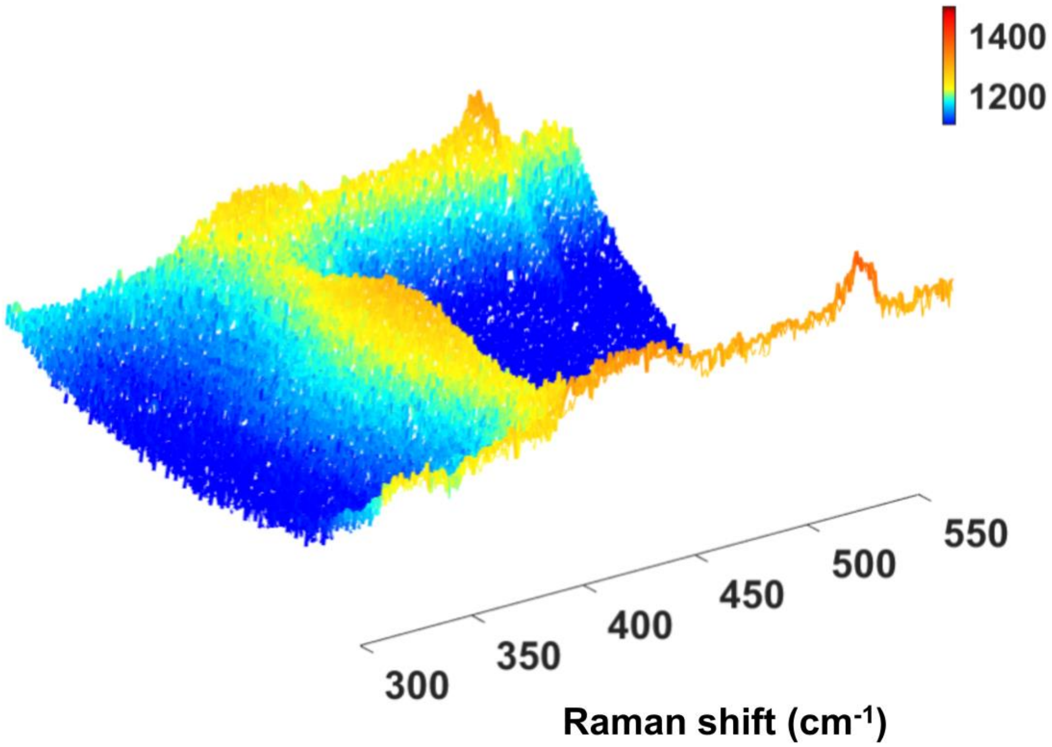

Figure S18. In-operando Raman spectrum during the charging-discharging process for the catholyte (10 mL of 0.5 M $Na_4[Fe(CN)_6]$).

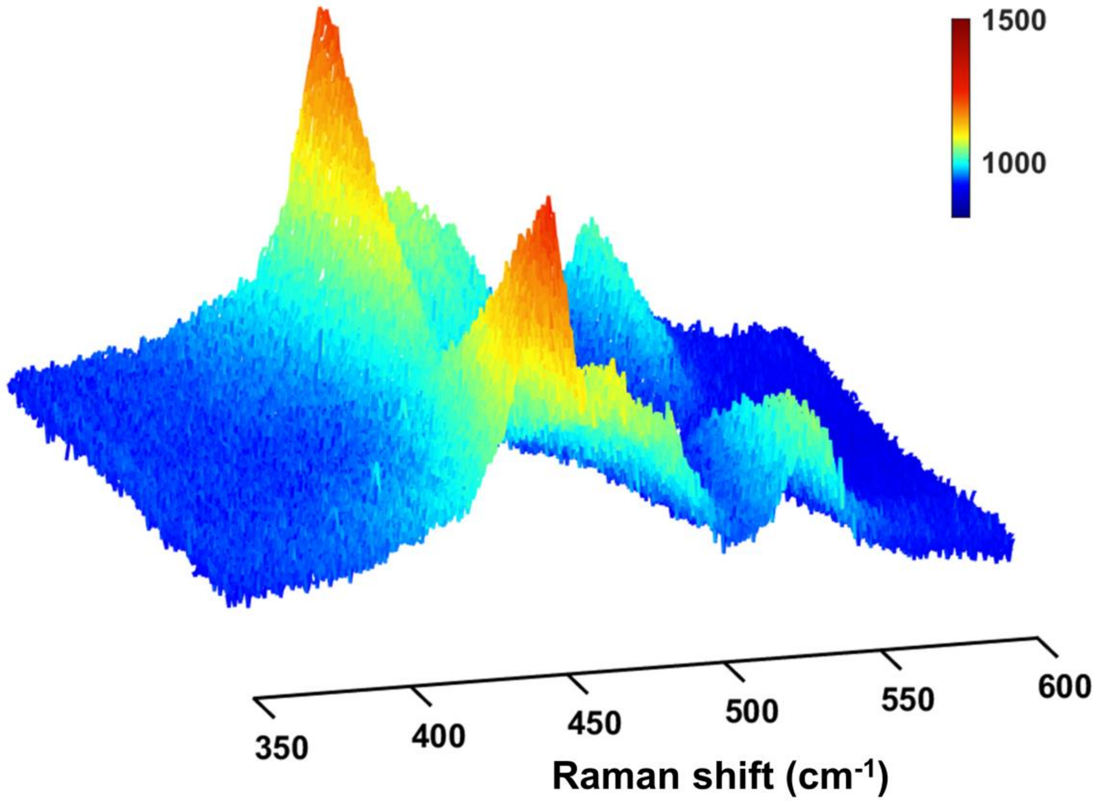

Figure S19. In-operando Raman spectrum during the charging-discharging process for the anolyte (11 mL of 0.25 M Na_2S_2 in 0.25 M Na_2SO_4, pH 13.5)

Reference

1. G. Kresse, J. Hafner, *Phys. Rev. B Condens Matter* **1993**, 47, 558-561.
2. G. Kresse, J. Furthmüller, *Phys. Rev. B* **1996**, 54, 11169-11186.
3. G. Kresse, D. Joubert, *Phys. Rev. B* **1999**, 59, 1758-1775.
4. J. P. Perdew, M. Ernzerhof, K. Burke, *J. Chem. Phys.* **1996**, 105, 9982-9985.
5. Perdew, J. P, Burke, K, Ernzerhof, M, *Phys. Rev. Lett.* **1996**, 77, 3865-3868.
6. S. Grimme, S. Ehrlich, L. Goerigk, *J. Comput. Chem.* **2011**, 32, 1456-1465.
7. H. J. Monkhorst, J. D. Pack, *Phys. Rev. B* **1976**, 13, 5188-5192.
8. G. Henkelman, A. Arnaldsson, H. Jonsson, *Comp. Mater. Sci.* **2006**, 36, 354-360.
9. K. Momma, F. Izumi, *J. Appl. Crystallogr.* **2011**, 44, 1272-1276.
10. Z. Li, G. Weng, Q. Zou, G. Cong, Y.-C. Lu, *Nano Energy* **2016**, 30, 283-292.
11. D. Ma, B. Hu, W. Wu, X. Liu, J. Zai, C. Shu, T. Tadesse Tsega, L. Chen, X. Qian, T. L. Liu, *Nat. Commun.* **2019**, 10, 3367.
12. Z. Li, Y.-C. Lu, *Nat. Energy* **2021**, 6, 517-528.
13. J. Zai, Y. Zhu, K. He, A. Iqbal, S. Huang, Z. Chen, X. Qian, *Mater. Chem. Phys.* **2020**, 250, 123143.
14. Y. Zhu, K. He, T. Tadesse Tsega, N. Ali, J. Zai, S. Huang, X. Qian, Z. Chen, *Sustain. Energ. Fuels* **2020**, 4, 2892-2899.
15. M. M. Gross, A. Manthiram, *Energy Storage Mater.* **2019**, 19, 346-351.
16. L. Su, A. F. Badel, C. Cao, J. J. Hinricher, F. R. Brushett, *Ind. Eng. Chem. Res.* **2017**, 56, 9783-9792.
17. M. M. Gross, A. Manthiram, *ACS Appl. Energ. Mater.* **2019**, 2, 3445-3451.
